# Systematic review of the impact of cannabinoids on neurobehavioral outcomes in preclinical models of traumatic and nontraumatic spinal cord injury

**DOI:** 10.1038/s41393-021-00680-y

**Published:** 2021-08-14

**Authors:** Faheem I. Bhatti, Oliver D. Mowforth, Max B. Butler, Aniqah I. Bhatti, Sylva Adeeko, Melika Akhbari, Rory Dilworth, Ben Grodzinski, Temidayo Osunronbi, Luke Ottewell, Jye Quan Teh, Sophie Robinson, Gayathri Suresh, Unaiza Waheed, Benn Walker, Isla Kuhn, Lara Smith, Richard D. Bartlett, Benjamin M. Davies, Mark R. N. Kotter

**Affiliations:** 1 Division of Neurosurgery, Department of Clinical Neurosciences, University of Cambridge, Cambridge, UK; 2 Myelopathy.org, Cambridge, UK; 3 Cambridge University Medical Library, Cambridge, UK

**Keywords:** Spinal cord diseases, Preclinical research

## Abstract

**Study design:**

Systematic review.

**Objectives:**

To evaluate the impact of cannabinoids on neurobehavioral outcomes in preclinical models of nontraumatic and traumatic spinal cord injury (SCI), with the aim of determining suitability for clinical trials involving SCI patients.

**Methods:**

A systematic search was performed in MEDLINE and Embase databases, following registration with PROPSERO (CRD42019149671). Studies evaluating the impact of cannabinoids (agonists or antagonists) on neurobehavioral outcomes in preclinical models of nontraumatic and traumatic SCI were included. Data extracted from relevant studies, included sample characteristics, injury model, neurobehavioural outcomes assessed and study results. PRISMA guidelines were followed and the SYRCLE checklist was used to assess risk of bias.

**Results:**

The search returned 8714 studies, 19 of which met our inclusion criteria. Sample sizes ranged from 23 to 390 animals. WIN 55,212-2 (*n* = 6) and AM 630 (*n* = 8) were the most used cannabinoid receptor agonist and antagonist respectively. Acute SCI models included traumatic injury (*n* = 16), ischaemia/reperfusion injury (*n* = 2), spinal cord cryoinjury (*n* = 1) and spinal cord ischaemia (*n* = 1). Assessment tools used assessed locomotor function, pain and anxiety. Cannabinoid receptor agonists resulted in statistically significant improvement in locomotor function in 9 out of 10 studies and pain outcomes in 6 out of 6 studies.

**Conclusion:**

Modulation of the endo-cannabinoid system has demonstrated significant improvement in both pain and locomotor function in pre-clinical SCI models; however, the risk of bias is unclear in all studies. These results may help to contextualise future translational clinical trials investigating whether cannabinoids can improve pain and locomotor function in SCI patients.

## Introduction

Spinal cord injury (SCI) is a traumatic event associated with severe disability and mortality [1]. Prevalence of SCI is estimated to be 906 cases per million in the United States and incidence as high as 58 cases per million per year in some European countries [2, 3]. The consequences of SCI encompass motor, sensory and autonomic domains [4]; functional disability, reduced quality of life and high prevalence of affective disorders are common [5]. Chronic neuropathic pain affects up to 75% of people with SCI [5]. Burning and shooting pain as well as hypersensitivity to cutaneous stimuli have detrimental effects on rehabilitation, mood and mental health [6].

The classic model of SCI consists of two phases [1]. The first phase involves direct damage as a result of mechanical trauma. This causes immediate damage and then catalyses the second phase of injury driven by aberrant molecular, cellular and biochemical cascades. Secondary injury constitutes damage caused by ischaemia, ionic derangements, excitotoxicity, free radical damage, oedema, inflammation and apoptosis [7, 8].

Cannabinoid (CB) receptor agonists are a promising pharmacological approach [9]. CBs were first identified as the psychoactive constituents of marijuana [10]. However, an endogenous cannabinoid system also exists, consisting of two CB receptors (CB1 and CB2), natural ligands (endo-CBs) such as anandamide, and enzymes involved in endo-CB synthesis and degradation [11]. Following SCI, local modulation of the endo-CB system has been reported [11]. This involves increased levels of anandamide, upregulation of the synthetic enzyme, and downregulation of the degradative enzyme. Moreover, the endo-CB system has been shown to be important in neuroprotection and immunomodulation after SCI [11], as well after cerebral ischaemia-reperfusion injury [12] and traumatic brain injury [13].

A number of cannabinoids have been shown to downregulate processes thought to be important in the secondary phase of SCI. For example, cannabidiol (CBD) is an exogenous cannabinoid receptor agonist currently being evaluated in a number of clinical trials for multiple medical conditions [14–17]. CBD has been shown to reduce reactive oxygen and nitrogen species production, chemokine and cytokine release, microglial and astrocyte activation, as well as T cell proliferation [18, 19]. In addition, WIN 55,212-2, a non-selective CB receptor agonist, downregulates central nervous systems neutrophil infiltration and apoptosis in multiple sclerosis [20], promotes neural remyelination in neonatal rats experiencing hypoxia-ischaemia [21] and relieves neuropathic pain following peripheral nerve injury in mice [22].

Given the mechanism of action of cannabinoids and the pathophysiology of SCI, there may be a therapeutic role for cannabinoids in patients following SCI. A recent systematic review of cannabinoid use in human patients with SCI found cannabinoid receptor agonists may be associated with reductions in pain and spasticity, however, the magnitude of these effects and clinical significance was unclear [23]. Furthermore, the overall quality of the included studies was reported as poor [23]. A series of reviews from the International Association for the Study of Pain (IASP) highlighted a similar lack of high-quality pre-clinical or clinical evidence for the use of cannabinoids in pain management specifically [24, 25]. This was the basis for the IASP position statement in March 2021, which stated that the IASP do not endorse the use of cannabinoids and cannabis-based medicine in pain management. Therefore, whilst cannabinoids have showed clinical promise, their clinical use remains limited by the strength of the pre-clinical and clinical evidence base.

This systematic review aims to evaluate the impact of cannabinoids, including cannabinoid receptor agonists, cannabinoid receptor antagonists, and endocannabinoid system modulators, on pain but also locomotor function and anxiolysis in preclinical models of SCI. Cannabinoid agonists are of interest firstly, due to their ability to downregulate processes involved in the inflammatory phase of SCI and secondly, due to reports of beneficial clinical effects following SCI. Furthermore, there exist several cannabinoid receptor agonists licensed for clinical use, and therefore evaluating the effects of cannabinoid agonists is important to gauge whether these drugs could potentially be used in translational clinical trials for patients who have undergone SCI. The effects of cannabinoid receptor antagonists and endocannabinoid system modulators are also of mechanistic interest, as they provide insight into the role of the endo-cannabinoid system following SCI.

This systematic review secondarily aims to offer discussion of the potential underlying mechanisms of action and the potential suitability of cannabinoids for future clinical trials in SCI patients. In this review, we also explore whether cannabinoids are associated with improvements in neurobehavioural outcomes in animal models of SCI and whether there may be merit in using cannabinoids to treat the symptoms experienced by patients following SCI.

The pressing unmet clinical need and scope for this review were identified through the REsearch Objectives and Common Data Elements for Degenerative Cervical Myelopathy (RE-CODE DCM) initiative, an international consortium of key stakeholders in degenerative cervical myelopathy (DCM), which provided consensus on DCM research priorities [26].

## Methods

This systematic review was conducted following the Preferred Reporting Items for Systematic Reviews and Meta-Analysis (PRISMA) guidelines. [27]

### Protocol and registration

The protocol for this review was published on PROSPERO (an international prospective register of systematic reviews) on 3 December 2019 (CRD42019149671).

### Eligibility criteria

The inclusion and exclusion criteria used in this review are presented in full in the Supplementary Material.

#### Population and injury model

This review included only preclinical animal models, and human studies were excluded. SCI models such as traumatic injury, spinal cord ischaemia or cryogenic spinal cord injury were included. Peripheral nerve injury or traumatic brain injury models were excluded.

#### Intervention and comparison

Studies were included if they used cannabinoid receptor agonists (e.g. WIN 55,212-2), cannabinoid receptor antagonists (e.g. AM251 or AM630) or endocannabinoid system modulators (e.g. acetaminophen or naloxone), administered intravenously, intraperitoneally or intrathecally. To be included, studies required a control treatment group and one or more cannabinoid treatment groups. Studies were not excluded based on drug administration parameters such as frequency or the duration of dosing.

#### Outcomes

Neurobehavioural outcomes, defined as outcomes of motor and/or sensory function, including pain, were the focus of this review. Studies that assessed neurobehavioural outcomes using tools such as the Basso, Beattie and Bresnahan score for locomotor function and von Frey filaments for mechanical allodynia and hyperalgesia were included. Studies that exclusively assessed other parameters, such as histological or autonomic outcomes, were excluded.

### Information sources

To identify articles, a systematic search was performed of MEDLINE and Embase databases on December 14, 2020.

### Search

The search strategy was developed with the assistance of a medical librarian at the Cambridge University Medical Library. The terms used to search MEDLINE and Embase are provided in the Supplementary Material. No additional search limits were applied.

### Study selection

Duplicates were excluded in Mendeley (Elsevier, London, UK). The abstracts were then screened independently by 14 of the authors using Rayyan software. The abstracts were divided into 7 groups. Each group of abstracts was reviewed by a pair of authors. Disagreements were resolved through discussion between the reviewers until mutual agreement was reached.

### Data extraction

The data extracted were: author, year of publication, country of experiments, study characteristics (e.g. number of experimental groups, level of evidence), sample characteristics e.g. size, number of groups, species, strain, age, sex, weight, and comorbidities), intervention (including injury model and the type, dose, frequency and route of drug), the methods and results of any neurobehavioural assessment, and the nature of any relevant statistical analysis performed. Data extraction was performed by one of the authors (FB).

### Analysis and reporting

Due to the diverse range of injury models, interventions and outcomes, meta-analysis was not possible and a narrative synthesis using the Synthesis Without Meta-analysis (SWiM) reporting guideline was conducted [28]. A checklist of adherence is provided in the Supplementary Material. Studies were primarily grouped based on outcome measures and secondarily the cannabinoid intervention used. The three broad outcome categories were motor function, pain and anxiolysis. The differences (mean/median/p values) between intervention and control groups reported by individual studies for each outcome measure was initially summarised in a table. Findings were subsequently summarised by transforming the difference measures into standardised metric-direction of effect (positive/negative/no effect) and synthesised in the form of harvest plots by vote-counting based on direction of effects. Combining p values and calculating summary statistics of intervention effect estimates was not possible. Heterogeneity in reported effects was investigated by structuring figures around the injury model, interventions, and outcomes assessed; these are provided in the Supplementary Material. The SYRCLE (Systematic Review Centre for Laboratory Animal Experimentation) tool was used to evaluate the risk of bias of the included studies [29]. Since risk of bias was determined to be ‘unclear’ for all the included studies, it could not be used to prioritise the reporting of certain findings over others, thus study findings were reported equally in the narrative synthesis.

## Results

### Study selection

**Fig. 1:**
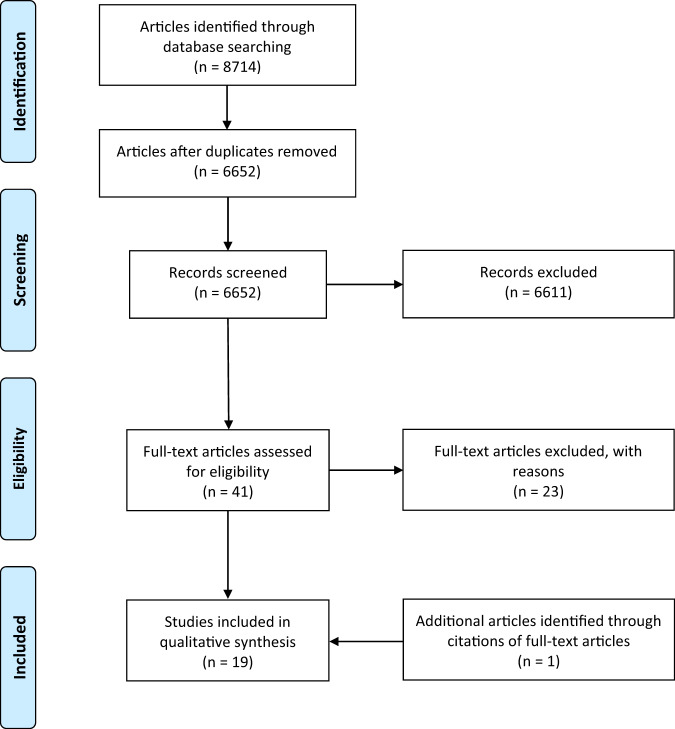
PRISMA flow diagram of study selection. Number of studies identified, screened, assessed for eligibility and included are shown.

The search generated 8714 results. A total of 2062 duplicates were removed using Mendeley (Elsevier, London, UK), resulting in 6652 unique studies. Following the abstract screening, 41 studies were found to meet the inclusion criteria (Supplementary Material). On full-text screening, 23 studies were excluded for the reasons outlined in the Supplementary Material. One additional relevant study was identified in the reference list of an included study. In total, 19 studies were included (Fig. 1).

### Study characteristics

Of the 19 included studies, 13 studies used rat models of SCI [30–42] and 6 used mice models [43–48]. Sprague-Dawley rats were used in 10 studies [30, 33–41] and Wistar rats in 3 studies [31, 32, 42]. With regards to strains of mice, CD1 mice were used by 3 studies [43, 45, 48] while CB57BL/6J [44], CB57BL/6 [46] and PPAR-*α*KO mice with litter-mate wildtype controls [47] were each used in 1 study. Male animals were used in 16 studies [30–45] whilst female animals were used in 1 [46]. 2 studies did not specify the sex of the animals used [47, 48]. The age of mice and rats were not specified in 11 studies [34–42, 46, 48]. Of the 8 studies which commented on animal age, 4 studies explicitly specified the age of the animals used [31, 33, 44, 47] and 4 studies stated adult animals were used [30, 32, 43, 45].

**Fig. 2:**
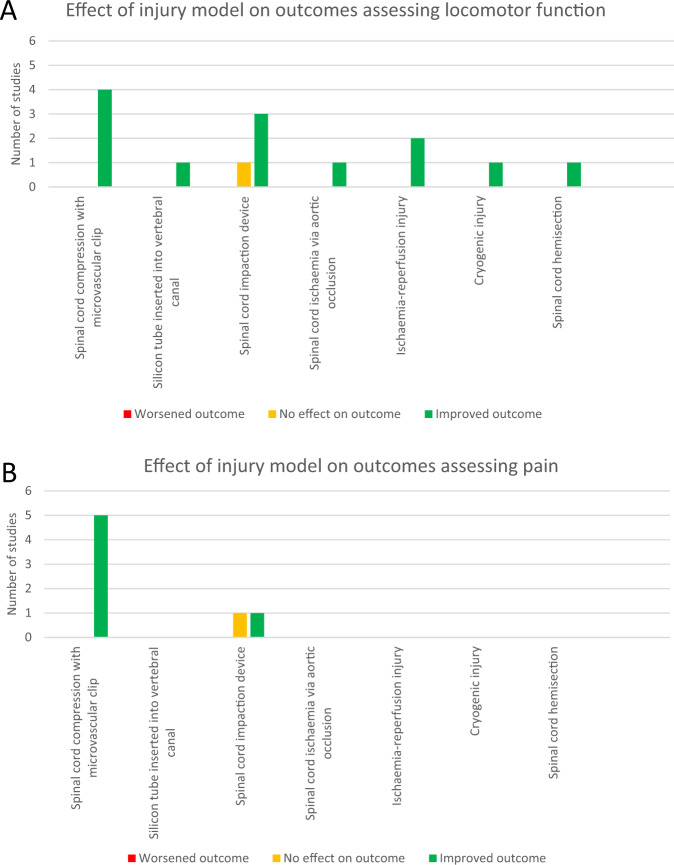
Visual representation of injury models used to assess locomotor function (A) and pain (B). Number of studies using each injury model and the reported effects on locomotor function (**A**) and pain (**B**) are shown.

The different injury models used are summarised in Fig. 2 and Table 1. Compression models, involving microvascular clips (*n* = 9) [35–39, 43, 45, 47, 48] and silicon tube insertion into the vertebral canal [44], and contusion models involving spinal cord impaction devices (*n* = 4) [30, 31, 33, 46] were the most commonly used. Other models used included spinal cord ischaemia via aortic occlusion [40], cryogenic injury using liquid nitrogen [42], spinal cord hemisection [32], ischaemia-reperfusion injury via aortic occlusion [34] and ischaemia-reperfusion injury via aortic clamping [41]. The majority of SCI models used thoracic injury (*n* = 15) [30, 31, 33, 35–39, 42–48], whilst one study used cervical level injury (*n* = 1) [32].

**Table 1:** Summary of injury models, advantages and limitations.

Injury model type	Description	Relevant methods	Advantages	Limitations
Compression	Prolonged spinal cord compression	• Microvascular clip [32–36, 43, 45, 47, 48] • Compression via silicon tube insertion into the vertebral canal [44]	• Mimics fracture dislocations and burst fractures [61]. • Relatively simple and inexpensive. • Can be used in different regions of spinal cord. • Clips of varying closing force are available to crudely vary severity of SCI. • Incorporates a degree of vascular occlusion and ischaemia [61].	• Difficult to standardise due to variability in the actual force applied by clips and extent of cord compression [61]. • Lacks acute impact phase of contusion models.
Contusion	Acute, blunt trauma to spinal cord.	• Infinite Horizons impactor device [31, 46] • New York University Weight drop device [41], • Multicenter Animal Spinal Cord Injury Study (MASCIS) weight-drop impactor [30]	• Controlled method of inducing SCI of defined severity [61]. • Representative of human SCI [51]. • Some devices allow real-time measurement of the force applied and allows sub-optimal injuries to be excluded [61].	• Requires specialist impactor equipment. • Often severe animal disability post-injury requiring significant aftercare. • Infinite Horizons: inconsistent injury due to difficulties stabilising spinal cord during impact [61]. • MASICS: Unable to control duration of impact. Risk of weight bounce causing multiple impacts [61].
Ischaemia / Ischaemia-Reperfusion.	Spinal cord hypoperfusion.	• Ischaemia: Aortic occlusion with balloon catheter [37] • Ischaemia-Reperfusion via aortic occlusion [42] or clamping [38]	• May be representative of ischaemia which can occur in surgery involving the aorta e.g. aneurysm repair [62].	• Varying compensatory supply from collateral arteries [63]. • Excludes any acute mechanical trauma and therefore may be of limited utility to human SCI. • Difficult to standardise as magnitude of SC damage in mouse models is influenced by strain, core temperature, body weight and plasma glucose levels [63].
Cryogenic	Localised thermal insult to spinal cord.	• Liquid nitrogen jet [39]	• Produces localised damage without affecting continuity of spinal cord [64].	• Not representative of injuries which cause human SCI.
Transection (partial)	Partial cut of spinal cord.	• Spinal cord hemisection [40]	• Most useful for investigating regeneration, degeneration and grafting [50]. • Allows comparison of deficit and recovery between injured and healthy tracts in the same animal providing intraanimal control [61]. • Consistent lesioning which can target specific anatomical tracts or regions.	• Pathophysiology may be less representative of human injuries which are most commonly contusive SCI [50]. • May not accurately recapitulate all aspects of the secondary injury cascade.

**Fig. 3:**
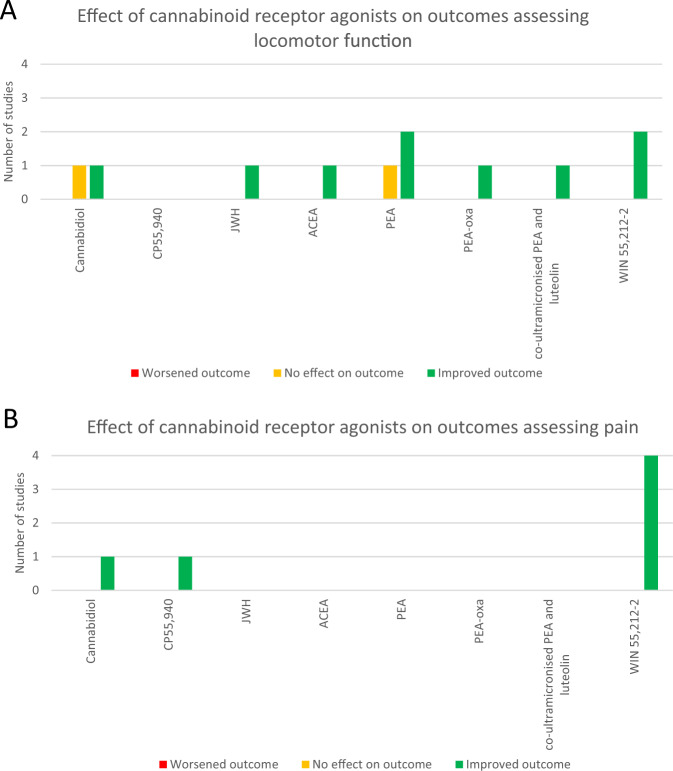
Visual representation of cannabinoid receptor agonists used to assess locomotor function (A) and pain (B). Number of studies using each cannabinoid receptor agonist and the reported effects on locomotor function (**A**) and pain (**B**) are shown.

Eight different cannabinoid receptor agonists were used as seen in Fig. 3. WIN 55,212-2 (*n* = 6) [30, 33, 35, 36, 38, 40] was the most commonly used agonist. Palmitoylethanolamide (PEA) [43, 47] and cannabidiol (CBD) [42, 46] were each used in 2 studies. CP 55,940 [39], JWH [32], N-(2-chloroethyl)−5Z, 8Z, 11Z, 14Z-eicosatetraenamide (ACEA) [44], oxazoline of Palmitoylethanolamide [45] and co-ultramicronised PEA and luteolin [48] were each used in 1 study. Two different inverse cannabinoid receptor agonists were used: rimonabant (*n* = 2) [38, 39] and hemopressin (*n* = 1) [38]. Five cannabinoid receptor antagonists were also used, with AM 630 (*n* = 8) [30, 31, 33–35, 37, 40, 41] and AM 251 (*n* = 7) [30, 33–35, 37, 40, 41] being the most commonly used. Other antagonists used included SR 144528 (SR 2) (*n* = 2) [32, 39] and AM 281(*n* = 1) [31]. One study used acetaminophen and naloxone [37]; both drugs have been reported to have effects on the endocannabinoid system. Initiation of cannabinoid administration ranged from 20 min [30] to 5 weeks [35] after SCI. The duration of cannabinoid administration ranged from single one-off doses to repeat dosing over 10 weeks [46]. A comprehensive summary of the drugs used in the included studies can be found in the Supplementary Material.

**Fig. 4:**
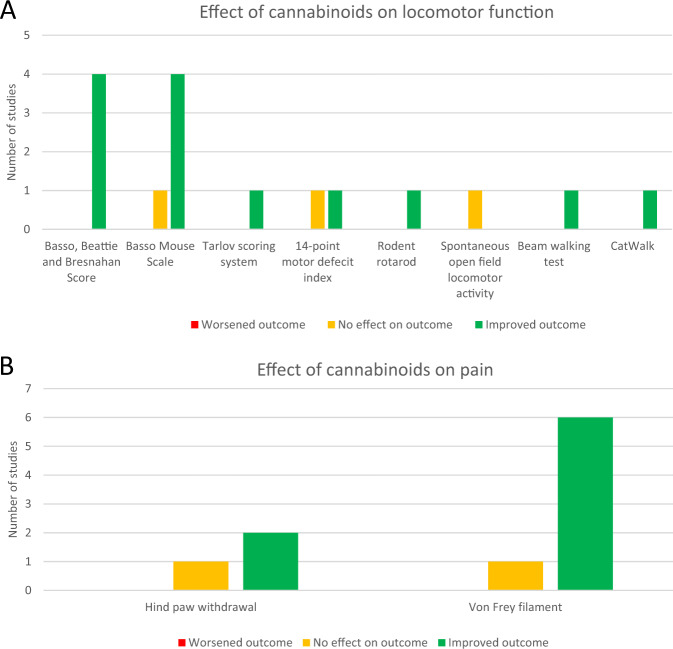
Visual representation of the effect of cannabinoids on locomotor function (A) and pain (B). Effect of cannabinoids on locomotor function (**A**) and pain (**B**) stratified by outcome measure is shown. Frequency of outcome measure use is also indicated.

Across the included studies, locomotor function was evaluated by 13 studies, pain perception was evaluated by 8 studies and 1 study explored anxiety. 8 different measures of locomotor function were used (summarised in Fig. 4A). The most commonly used measures of locomotor function were Basso Mouse Scale (BMS) (*n* = 5) [44–48], Basso, Beattie, Bresnahan (BBB) Locomotor score (*n* = 4) [31, 33, 42, 43] and 14-point motor deficit index (MDI) score (*n* = 2) [34, 40]. Beam-walking test [32], CatWalk [32], rodent rotarod) [44], spontaneous open-field locomotor activity [44] and Tarlov scoring system [41] were each used once. Two measures of pain were used (summarised in Fig. 4B), namely the von Frey filament test (*n* = 5) [31, 35–39] and hind paw withdrawal to thermal stimulus (*n* = 3) [30, 31, 46]. The elevated plus-maze test [44] was the sole measure of anxiety (*n* = 1). No studies assessed the sedative effects of cannabinoids. Table 2 summarises all of the scoring systems used to evaluate outcomes. Assessment time points ranged from 30 min [35] to 90 days [31] after cannabinoid administration. Table 3 groups the included studies by neurobehavioural outcome, and summarises the sample features, injury models, interventions, assessment tools and key findings. Figs 2 and 3 investigate the effects of specific cannabinoid interventions and injury model on locomotor and pain, and highlight the heterogeneity of the included studies. Fig. 4 visually represents the overall effects of cannabinoids on locomotor function and pain. Further information relating to individual studies can be found in the Supplementary Material.

**Table 2:** Summary of functional outcome tools used in included studies.

Outcome	Scales	Summary of tool
Locomotor	Basso, Beattie, Besnahan (BBB) Locomotor [27, 29, 30, 41]	Assesses hindlimb movement, paw placement, weight bearing, trunk stability, tail position and limb coordination. Scored from 0 to 21; 0 is no hindlimb movement, 21 is normal function.
Locomotor	Basso Mouse Scale (BMS) [28, 36–39]	Assesses the severity of SCI-induced paralysis based on hindlimb movement. Scored from 0 to 9.
Locomotor	Rodent rotarod [39]	Assesses vestibulomotor function using an accelerating rotarod and measuring latency time for mice to fall.
Locomotor	Spontaneous open field locomotor activity [39]	Assesses locomotor activity using a computerised video capture system.
Locomotor	14-point motor deficit index (MDI) score [40, 43]	Assesses motor function deficits in the hind limbs using a 14-point score. 0 is normal motor function, 14 indicates no movement and dragging of hind limbs.
Locomotor	Beam-walking test [42]	Assesses fine-motor behaviour using an elevated narrow beam. Scored from 0 (rat unable to traverse the beam) to 6 (rat traversed beam normally).
Locomotor	CatWalk [42]	Assesses locomotor gait dynamics using the catWalk gait analysis system (version 8.1; Noldus, Wageningen, The Nederlands).
Locomotor	Tarlov scoring system [44]	Assess neurological function on a scale of 0 (no lower extremity function) to 4 (normal motor function).
Pain	von Frey filament test [27, 28, 31–35]	Assess sensitivity to innocuous mechanical stimulation using von Frey filaments. The smallest filament that elicits a response is recorded.
Pain	Hind paw withdrawal to thermal stimulus [26–28]	Assesses sensitivity to thermal stimulation (hot or cold). Withdrawal latency time is measured.
Anxiety	Elevated plus maze test [39]	Assesses anxiolysis using an elevated platform with two open arms and two closed arms. Number of open arm and close arm entries are recorded.

**Table 3:** Summary of all included studies.

Author (Year)	Animal used	Number of animals	Injury model	Level of injury	Cannabinoid receptor agonists used	Cannabinoid receptor antagonists or inverse agonists used	Dose escalation study?	Outcomes assessed	Time of assessment	Key findings
Genovese et al. (2008) [43]	CD1 mice	30	Traumatic SCI Compression of spinal cord for 1 min using aneurysm clip (24 g)	T5-T8	• PEA	• None	• No	• BBB score	• Once a day for 10 days after SCI.	• PEA pre- or post-treatment reduced functional deficits (*p* < 0.05).
Kwiatkoski et al. (2012) [39]	Wistar rats	28	Cryogenic SCI Liquid nitrogen jet applied to spinal cord for 5 s	T10	• CBD	• None	• No	• BBB Score	• Before surgery and day 1, 3 and 7 after surgery	• CBD treatment improved locomotor function (*p* < 0.05).
Su et al. (2017) [41]	Sprague-Dawley rats	50	Traumatic SCI 10 g impactor dropped from 25 mm.	T9–10	• WIN	• AM 251 • AM 630	• No	• BBB Score	• Day 1, 3, 7, 14, 21 and 28 after SCI.	• WIN improved BBB scores (*p* < 0.01). • AM 630, but not AM251, abrogated the BBB score improvement by WIN (*p* < 0.01).
Impellizzeri et al. (2017) [45]	CD1 mice	80	Traumatic SCI Compression of spinal cord for 1 min using microaneurysm clip (24 g)	T6–7	• PEA-OXA	• None	• No	• Basso Mouse Scale	• Daily for 10 days after SCI.	• PEA-OXA reduced functional deficits induced by SCI (*p* < 0.05). • No significant difference between intraperitoneal or oral administration of PEA-OXA.
Paterniti et al. (2013a) [47]	PPAR-*α*KO mice (homozygous for the Ppara^tm1Gonz^ targeted mutation, strain name: 129S4/SvJae-Ppara^tm1Gonz^/J) and litter-mate	390	Traumatic SCI Compression of spinal cord for 1 min using microaneurysm clip (24 g)	T6–7	• PEA	• GW9662 • GSK0660	• No	• Basso Mouse Scale	• Daily for 10 days after SCI.	• PEA improved BMS scores (*p* < 0.05). • GSK0660 and GW9662 abolished the effect of PEA • Genetic absence of the PPAR-*α* receptor in PPAR-*α*KO mice blocked the effect of the PEA.
Paterniti et al. (2013b) [48]	CD1 mice	50	Traumatic SCI Compression of spinal cord for 1 min using microaneurysm clip (24 g)	T6–7	• PEA • PEA + Luteolin • Co-ultramicronized composite of PEA and Luteolin (co-ultraPEALut)	• None	• No	• Basso Mouse Scale	• Daily for 10 days after SCI.	• PEA, luteolin or a combination of PEA and luteolin had no effect on functional deficits. • Co-ultraPEALut reduced locomotor deficits (*p* < 0.01).
Latini et al. (2014) [40]	Wistar rats	40	Traumatic SCI Lateral cervical spinal cord hemisection (SCH) using iridectomy scissors.	C4	• JWH-015	• SR2	• No	• Beam-walking test • catWalk	• Beam walking: Day 0 (control), 1, 3, 5, 7, 21 and 60 days after surgery. • catWalk: Day 7, 21, 60	• JWH-015, but not SR2, improved locomotor function as assessed by beam walking and catWalk (*p* < 0.001).
Jing et al. (2017) [38]	Sprague-Dawley rats	168	Ischemia/Reperfusion Injury Cross-clamp applied to aortic arch (between left common carotid artery and left subclavian artery) for 14 min.	N/A	• None	• AM 251 • AM 630	• No	• Tarlov scoring system	• 4 h and 24 h after reperfusion.	• AM 251 and AM 630 both abolished the neuroprotective effects of remote ischaemic preconditioning prior to SCI (*p* < 0.05).
Huo et al. (2018) [37]	Sprague-Dawley rats	40	Spinal cord ischaemia Catheter occlusion (catheter tip was at the level of the subclavian artery of aorta) for 12 min.	N/A	• WIN	• AM 251 • AM 630	• No	• 14-point motor deficit index (MDI) score	• 48 h after reperfusion	• WIN improved MDI scores compared to control group (Control 5 [2], WIN 55,212-2 2.5[1.25]; *p* < 0.05). • AM 630, but not AM 251 reduced the effect of WIN on MDI scores (WIN 55,212-2 2.5[1.25], AM 630 5.2[1.25]; *p* < 0.01). • (Data expressed as median [interquartile range])
Su et al. (2009) [42]	Sprague-Dawley rats	64	Ischemia/Reperfusion Injury Aortic occlusion with Fogarthy catheter (catheter tip was at level of left subclavian artery) for 12 min	N/A	• None	• AM 251 • AM 630	• No	• 14-point motor deficit index (MDI) score	• 24 and 48 h after reperfusion.	• AM 251, but not AM 630, abrogated the protective effects of remote ischaemic preconditioning prior to SCI (*p* < 0.05).
Hong et al. (2015) [44]	C57BL/6J mice	53 (one mouse in the vehicle group died so only 52 survived the full 21 day experimental period.	Traumatic SCI 1.5 mm silicon tube placed into the T11 vertebral canal for 60 min	T11	• ACEA	• None	• No	• Rodent rotarod • Basso Mouse scale • Spontaneous open field locomotor activity • Elevated plus maze test (looking at anxiolytic effects	• Rodent rotarod: 1 day prior to and 1, 2, 7, 14, and 21 days after injury. • Basso Mouse scale for locomotion: 21 days after injury. • Spontaneous open field locomotor activity: 1, 2, 4, 24 h post treatment. • Elevated plus maze test: after 2 weeks of treatment.	• ACEA improved rotarod function (*p* = 0.04) and BMS scores (ACEA = 8 ± 3, vehicle = 5 ± 6; *p* = 0.02). • ACEA did not affect spontaneous activity or anxiolysis.
Arevalo-Martin et al. (2012) [31]	CD1 mice	35	Traumatic SCI Contusive injury using 200Kdyn for 5 seconds	T8	• None	• AM 281 • AM 630	• No	• BBB Score • Hind paw withdrawal from hot and cold plates. • von Frey filament test	• BBB Score: day 7, 14, 30, 60, 90 post SCI. • Hind paw withdrawal to a noxious thermal stimulus: tested twice with a 30 min interval, on day 60 and 90 post SCI.	• BBB Locomotor Scale: AM 281 and AM 630 reduced recovery in motor function (*p* < 0.05) • Hind paw withdrawal to noxious thermal stimulus: AM 281 and AM 630 had no notable effect. • Von Frey Filament test: AM 281 and AM 630 had no notable effect.
Li et al. (2018) [46]	C57BL/6 mice	25	Traumatic SCI Contusive injury by impactor drop from 3mm to deliver 60 kdyn force to spinal cord.	T9–10	• CBD	• None	• No	• Basso Mouse Scale • Hindpaw withdrawal from thermal stimulus • von Frey filaments test	• Basso Mouse Scale: Day 1, 3, 7 and thereafter once weekly for 10 weeks • Hindpaw withdrawal from thermal stimulus: before surgery then week 4, 6, and 10 after SCI. • Von Frey filaments test: before surgery then week 2, 4, 6, 8, 10 after SCI.	• CBD had no effect on locomotor function assessed using the Basso Mouse Scale. • CBD reduced thermal (*p* < 0.0001) and mechanical (*p* = 0.04) hypersensitivity.
Ahmed et al. (2010) [30]	Sprague-Dawley rats	91–99 (unclear)	Traumatic SCI Contusive spinal cord injury using 10 g rod dropped from height of 12.5 mm	T9	• WIN	• AM 251 • AM 630	• Yes	• Hind paw withdrawal to noxious thermal stimulus	• Pre-SCI. • Day 21, 28, 35, 42 post SCI, 45 min after drug administration.	• WIN 55,212-2 produced dose-dependent reduction in thermal hyperalgesia (*p* < 0.05). • Effect abolished by antagonist AM 630 but not AM 251.
Hama et al. (2007) [32]	Sprague-Dawley rats	48	Traumatic SCI Compression of spinal cord for 1 min using microaneurysm clip (24 g)	T6-T8	• WIN	• AM 251 • AM 630	• Yes	• von Frey filament test	• Baseline taken after SCI. • Agonist Experiment: Once every 30 min for 120 min following injection. • Agonist + Antagonist Experiment: 30 min after injection of agonist and antagonist	• WIN produced dose-dependent reduction withdrawal thresholds (WIN 55,212-2 0.2 mg/kg increased hindpaw withdrawal latency from 9.5 ± 0.4 s to 11.1 ± 0.5 s on day 42 post injury; *p* < 0.05. WIN 55,212-2 2 mg/kg increased hindpaw withdrawal latency from 8.4 ± 0.4 s to 10.7 ± 0.4 s on day 42 post injury; *p* < 0.0001) • Effect blocked by AM 251 but not AM 630 (withdrawal threshold after AM 630 pre-treatment = 9.9 ± 0.6 s, after WIN 55–212,2 post-treatment = 8.6 ± 1.0 s; *p* > 0.05. After-AM 251 pre-treatment 9.7 ± 0.6 s, after WIN 55–212,2 post-treatment 12.0 ± 0.7 s; *p* < 0.0001)
Hama et al. (2009) [33]	Sprague-Dawley rats	23	Traumatic SCI Compression of spinal cord for 1 min using microaneurysm clip (20 g)	T6–7	• WIN	• None	• Yes	• von Frey filament test	• Baseline taken before SCI, then before and 30 min after each injection of WIN for 7 days	• WIN produced dose-dependent reduction withdrawal thresholds (*p* < 0.05) • Effect was maintained throughout the 7 day experimental period.
Hama et al. (2010) [34]	Sprague-Dawley rats	*n* = 452–488 (Unclear)	Traumatic SCI Compression of spinal cord for 1 min using microaneurysm clip (20 g)	T6–7	• Acetaminophen (APAP) • Gabapentin • Memantine hydrochloride • Memantine sulphate • Morphine • Tramadol	• AM 251 • AM 630 • Naloxone	• Yes	• von Frey filament test	• Baseline taken 4 weeks after spinal cord compression. • Experiment 1: Every 30 min up to 120 min post-injection. • Experiment 2: 60 or 90 min after the second injection depending on the particular combination.	• APAP + Gabapentin combination demonstrated synergy (*p* < 0.05). Reduction in withdrawal thresholds was attenuated by AM 251 but not AM 630 (*p* < 0.05). • APAP + Memantine combination was merely additive. • APAP + Morphine combination demonstrated synergy (*p* < 0.05). Reduction in withdrawal thresholds was attenuated by AM 251 and AM 630 (*p* < 0.05). • APAP + Tramadol combination was merely additive. Reduction in withdrawal thresholds was attenuated by AM 251 and naloxone but not AM 630 (*p* < 0.05). • AM 251, AM 630 and naloxone did not affect withdrawal thresholds when given alone or prior to individual drugs.
Hama et al. (2011) [35]	Sprague-Dawley rats	198–206 (unclear)	Traumatic SCI Compression of spinal cord for 1 min using microaneurysm clip (20 g)	T6–7	• WIN	• Hemopressin • Rimonabant	• Yes	• von Frey filament test	• Single drug experiment: Prior to drug administration and every 30 min up to 2 h post injection. • Drug combination experiment: Prior to, and 30 min after WIN treatment.	• Intrathecal WIN 57.4 nmol increased withdrawal thresholds (*p* < 0.05). Lower doses had no effect. • Intracerebroventricular WIN produced a dose-dependent increase in withdrawal thresholds (*p* < 0.05). • Rimonabant and hemopressin did not alter withdrawal thresholds when used singly. • Rimonabant, but not hemopressin, blocked the antinociceptive effects of WIN (*p* < 0.05).
Hama et al. (2014) [36]	Sprague-Dawley rats	120	Traumatic SCI Compression of spinal cord for 1 min using microaneurysm clip (20 g)	T6–7	• CP 55,940	• Rimonabant • SR 144538	• Yes	• von Frey filament test	• Experiment 1 (Acute dose of CP 55,940): Before injection, every 30 min up to 120 min post injection. • Experiment 2 (Repeat drug treatment): After 8 am injection for 7 days. • Experiment 3 (Antagonist pre-treatment before CP 55,940 administration): Before pre-treatment and 30 and 60 min after treatment.	• Experiment 1: CP 55,940 produced dose-dependent increase in withdrawal thresholds (*p* < 0.05). • Experiment 2: Antinociceptive effects of CP 55,940 was maintained at full efficacy throughout 7 day experimental period. • Experiment 3: Pre-treatment with rimonabant, but not SR 144528 blocked the effect of CP 55,940 (*p* < 0.05).

### Risk of bias

Allocation sequence was adequately generated and applied in 12 out of 19 studies, the remaining studies may have been randomised but did not describe their allocation sequence. Four studies stated that group neurobehavioural characteristics were similar to baseline. No studies stated if the allocation was adequately concealed or if animals were randomly housed during the experiment. No studies stated if animals were selected at random for outcome assessment. The outcome assessor was blinded in 7 out of 19 studies; however, no studies explicitly stated that caregivers or investigators were blinded. One study stated one mouse in the vehicle group died but provided no further information regarding under what circumstances this occurred. A lack of reporting of items on the SYRCLE checklist across the 19 included studies means that accurately determining the risk of bias is challenging. This means that the overall risk of bias remains unclear and all results must be interpreted in the context of this. Full details of risk of bias assessment are provided in the Supplementary Material.

### What is the impact of cannabinoids on locomotor outcomes?

13 studies assessed locomotor outcomes of which five assessed BMS [44–48], four assessed BBB locomotor score [31, 33, 42, 43], two assessed 14-point MDI [34, 40]. Rodent rotarod [44], spontaneous open-field locomotor activity [44], beam walking [32], tarlov score [41] and catWalk assessment [32] were each used in one study.

#### Basso Mouse Scale (BMS) score

Five studies assessed BMS score of which one used ACEA [44], one used CBD [46], two used PEA [47, 48] and one used PEA-OXA [45].

Hong et al. observed that ACEA treated mice had higher BMS scores at 21 days post-SCI compared to vehicle (ACEA = 8 ± 3, vehicle = 5 ± 6, *p* = 0.02) [44]. By 3 weeks post-SCI most mice in both ACEA and vehicle-treated groups were able to use hindlimbs to support body weight (BMS score >5) [44]. However, a  greater number of mice were able to co-ordinate forelimb, hindlimb and tail function (BMS = 9) in the ACEA treated group (5 of 12) compared to the vehicle-treated group (0 of 11) (*p* = 0.02) [44].

CBD treatment was not shown to produce a significant effect on BMS scores compared to vehicle-treated mice over a period of 10 weeks post-SCI in the study by Li et al. [46].

Of the two studies using PEA [47, 48], 1 used PEA alone and in combination with PPAR-*γ* and PPAR-*δ* antagonists GW9662 and GW0660 [47] in wild type (WT) and PPAR-*α* knock-out (PPAR-*α*KO) mice. The other used PEA alone, PEA associated with luteolin (flavindoid with antioxidant properties) and a co-ultramicronised composite of PEA and luteolin [48] in WT mice. Both studies induced SCI using microvascular clips and recorded BMS scores daily for 10 days post-SCI. Treatment of WT mice with i.p. PEA (10 mg/kg) exclusively produced a significant increase in BMS scores in one study (*p* < 0.05) [47]. Pre-treatment with GSK0660 or GW9662 abolished the PEA-induced increase in BMS score [47]. Similarly the genetic absence of the PPAR-*α* receptor blocked the effect of PEA treatment [47]. In the other study, treatment of WT mice with i.p. PEA (1mg/kg) did not produce a significant increase in BMS scores. Treatment with PEA associated with luteolin also did not improve BMS scores compared to vehicle-treated control mice (*p* > 0.05) however, treatment with the co-ultramicronised composite of PEA and luteolin significantly reduced motor disturbance after SCI(*p* < 0.01) [48].

Using PEA-OXA, Impellizzeri et al. found treatment with 10 mg/kg significantly improved BMS scores post-SCI compared to vehicle-treated mice, and the effect was maintained until the end of the 10-day post-SCI experimental period (*p* < 0.05) [45].

#### Basso, Beattie, Besnahan (BBB) Locomotor Score

Of the four studies that assessed BBB score, one used PEA [43], one used WIN 55,212-2 alone and in combination with AM 251 or AM 630 [33], one used AM 251 and/or AM 630 [31], and one used CBD [42].

Genovese et al. found pre- or post-treatment with PEA significantly reduced the functional deficits induced by SCI over a 10-day period following SCI (*p* < 0.05 compared to vehicle-treated controls) [43]. No significant difference was found between PEA administered as a pre- or post-treatment [43]. WIN 55,212-2 was shown to promote functional recovery, measured by BBB score, following SCI [33]. BBB scores of WIN 55,212-2 treated animals continued to improve until 3 weeks following SCI at which point they scored more than 12, whereas BBB scores of control animals plateaued 1 week post-SCI (*p* < 0.0001) [33]. Significant differences were noted between WIN 55,212-2 and control-treated animals from day 7 to day 28 post-SCI (*p* < 0.01 at each individual timepoint) [33]. Pre-treatment with AM 630 (CB2 R antagonist) but not AM 251 (CB1 R antagonist) reversed the improvement induced by WIN 55,212-2 treatment (*p* = 0.0001 and *p* = 0.1879 respectively) [33]. In the study by Kwiatkoski et al. control rats and CBD treated rats both scored zero on the first day after SCI [42]. However, on day 3 and 7, CBD treated rats obtained higher BBB scores compared to controls (Day 3, CBD treated rats median = 2, vehicle-treated rats median = 0.5; *p* < 0.05. Day 7, CBD treated rats median = 7, vehicle-treated rats median = 4.5; *p* < 0.05) [42].

Using the selective CB1 R and CB2 R antagonists, AM 281 and AM 630, Arevalo-Martin et al. found the no difference between the BBB scores of cannabinoid antagonist and vehicle-treated mice at day 7 and 14 post-SCI [31]. At day 30, 60 and 90 cannabinoid antagonist treated mice scored significantly lower that vehicle-treated mice (Day 30: AM630; *p* < 0.05, AM281 + AM630; *p* < 0.05. Day 60: AM281; *p* < 0.05, AM630; *p* < 0.05, AM281 + AM630; *p* < 0.001. Day 90: AM281; *p* < 0.05, AM630; *p* < 0.05, AM281 + AM630; *p* < 0.05), with antagonist treated mice reaching a plateau at day 30 compared to vehicle-treated mice which continued to improve until day 60 [31].

#### The 14-point motor deficit index score

Two studies assessed the 14-point motor deficit (MDI) score [34, 40]. Huo et al. studied the effects of WIN 55,212-2 alone and alongside AM 251 or AM 630 following spinal cord ischaemia [40]. Su et al. investigated the effects of CB1 and CB2 receptor antagonists, AM 251 and AM 630, on the ischaemic tolerance induced by remote ischaemic preconditioning (RIPC) prior to ischaemia/reperfusion injury [34].

Following spinal cord ischaemia, WIN 55,212-2 treatment was observed to reduce the MDI score compared to control treatment (WIN 55,212-2 median = 2.5, interquartile range (IQR) = 1.25, control median = 5, IQR = 2; *p* < 0.05) [40]. This improvement was noted to be reversed by CB2 receptor antagonism with AM 630 but not by CB1 receptor antagonist with AM 251 (WIN 55,212-2 median = 2.5, IQR = 1.25, WIN 55,212-2 + AM630 median = 5, IQR = 1.25; *p* < 0.01, WIN 55,212-2 + AM251 median = 2.5, IQR = 1.25; *p* > 0.05) [40].

Su et al. observed no difference between the MDI scores of rats treated with RIPC, AM 630 + RIPC, and vehicle + RIPC. The MDI scores of rats treated with RIPC was significantly lower than AM 251 + RIPC treated rats (*p* < 0.05). No significant difference was recorded between the MDI scores of rats treated with AM251 + RIPC, AM 251, AM 630 or vehicle.

#### Rodent rotarod

One study assessed rodent rotarod performance [44]. Hong et al. found ACEA (3 mg/kg/day) treatment improved rotarod function over the 21 day recovery interval compared to vehicle treatment (*p* = 0.04) [44].

#### Spontaneous open field locomotor activity

One study assessed open field locomotor activity of mice treated with ACEA. ACEA was not found to affect spontaneous activity post-SCI [44].

#### Beam-walking test

Latini et al. found rats treated with either JWH-015, SR2, or saline after lateral cervical spinal cord hemisection all displayed uniform severe motor impairments at day 1 post-SCI [32]. From day 3 post-SCI onwards, rats treated with JWH-015 had better beam-walking scores than saline (*p* < 0.001) and SR2 (*p* < 0.0001) treated rats [32]. No differences in score were observed between rats treated with SR2 or saline [32].

#### CatWalk

Using CatWalk video analysis, Latini et al. found all rats treated with either JWH-015, SR2 or saline to display clear deficits in all parameters on day 7 post-SCI [32]. However, from day 7 to day 60 post-SCI, JWH-015 treated rats achieved significantly better print length (*p* < 0.001), print width (*p* < 0.0001), print area (*p* < 0.001), regularity index (*p* < 0.0001) and maximum contact area (*p* < 0.001) than both SR2 and saline-treated rats [32]. No differences were observed between saline and SR2 treated rats [32].

#### Tarlov scoring system

One study assessed neurological function using the Tarlov scoring system [41]. Jing et al. studied the effect of AM 251 and AM 630 pre-treatment on the neuroprotective effects of RIPC prior to ischaemia/reperfusion injury [41]. At 4 h after reperfusion, AM 251 pre-treatment + RIPC treated rats achieved significantly lower Tarlov scores compared to rats subject to RIPC only (*p* < 0.05) [41]. The scores of RIPC only, AM630 pre-treatment + RIPC, and vehicle pre-treatment + RIPC treated mice were not significantly different at this time point [41]. At 24 h post-reperfusion, both AM 251 and AM 630 pre-treatment statistically abolished the neuroprotective effect of RIPC as measured by Tarlov scores (*p* < 0.05) [41].

### What is the impact of cannabinoids on pain outcomes?

Eight studies assessed pain outcomes of which seven used the von Frey filament test [31, 35–39, 46] and three assessed hind paw withdrawal thresholds [30, 31, 46].

#### Von Frey filament test

Of the seven studies assessing mechanical sensitivity using the von Frey filament test, one study used CBD [46], three used WIN 55,212-2 [35, 36, 38], one used CP 55,940 [39], one used hemopressin [38], two used rimonabant [38, 39], one used AM 251 [35, 37], one used AM 281 [31], three used AM 630 [31, 35, 37], and one used SR 144528 [39]. One study used acetaminophen either alone or in combination with gabapentin, memantine, morphine or tramadol [37].

The study by Li et al. found no significant main effect of CBD treatment on right hindpaw von Frey filament scores [46]. Changes in mechanical sensitivity were noted to be variable between mice and between the left and right paw of individual mice, with both increases and decreases in sensitivity observed [46].

Of the two studies that administered WIN 55,212-2 subcutaneously, one administered WIN 55,212-2 as a single dose at 4–5 weeks post-SCI [35] and one administered WIN twice daily for 7 days beginning 4 weeks post-SCI [36]. Both studies found WIN-55,212-2 increased withdrawal thresholds in a dose-dependent manner (*p* < 0.05 in both studies) [35, 36]. This was observed for 2 h following single WIN 55,212-2 treatment by Hama et al. [35] and 30 min after each WIN 55,212-2 injection by Hama et al. [36]. The antinociceptive effect of WIN was maintained throughout the duration of the 7 day experimental period in the Hama et al.(2009) study [36]. Pre-treatment with AM 251 but not AM 630 was observed to block the antinociceptive effect of subcutaneous WIN 55,212-2 [35].

Hama et al. [38], investigated the effects of centrally mediated CB receptor ligands following SCI [38]. Intrathecal WIN 55,212-2 (57.4 nmol) significantly increased withdrawal thresholds from 30 min post-administration to 120 min post-administration (*p* < 0.05). Lower doses of intrathecal WIN 55,212-2 were not reported to affect withdrawal thresholds [38]. Intracerebroventricular WIN 55,212-2 produced a dose-dependent antinociceptive effect at 30 min post-administration, but not at any other time point (*p* < 0.05 at 30 min) [38].

Intrathecally injected hemopressin and rimonabant did not significantly alter hind paw withdrawal threshold [38]. Hemopressin administered either as a pre-treatment intrathecally or as an intracerebroventricular co-treatment with WIN 55,212-2 did not alter the antinociceptive effect of intrathecal WIN 55,212-2 [38]. By contrast intrathecal rimonabant pre-treatment blocked the antinociceptive effect of intrathecal WIN 55,212-2 (*p* < 0.05 compared to vehicle pre-treatment) [38]. Subcutaneous pre-treatment with rimonabant blocked the antinociceptive effect of intracerebroventricular WIN 55,212-2 (*p* < 0.05 compared to vehicle pre-treatment) [38].

Arevalo-Martin et al. observed no difference between rats treated with AM 281 (3 mg/kg), AM 630 (3 mg/kg) or a combination AM 281 (3 mg/kg) and AM 630 (3 mg/kg), and rats treated with vehicle following SCI [31].

Hama et al. [39] found CP 55,940 increased withdrawal threshold compared to vehicle in a dose-dependent manner (*p* < 0.05) [39]. Peak efficacy was not observed until 60–90 min following injection of lower doses (0.03 and 0.1 mg/kg) but was observed rapidly following injection with the highest dose (0.3 mg/kg) [39]. Hama et al. [39] noted the antinociceptive effect of CP 55,940 was maintained at full efficacy with twice-daily dosing over a 7-day observation period [39]. Pre-treatment with the CB1 receptor antagonist rimonabant (*p* < 0.05) but not the CB2 receptor antagonist SR 144528 (*p* > 0.05) blocked the effect of CP 55,90 [39].

Hama et al. [37] studied the effects of using acetaminophen alone or in combination with other analgesics on mechanical sensitivity, measured using the von Frey filament score, and presented as a percent maximum possible effect [37]. The maximum possible effect of acetaminophen (100 mg/kg) at 60 and 90 min post-SCI were not significantly different to that of vehicle-treated rats [37]. Combinations of acetaminophen with either gabapentin or morphine produced statistically significant synergy (*p* < 0.05 compared to effects of drugs used individually) [37]. Combinations of acetaminophen with either memantine or tramadol did not produce any statistically significant synergy (*p* > 0.05 compared to effects of drugs used individually) [37]. Pre-treatment with AM 251 but not AM 630 significantly attenuated the antinociceptive effect of the acetaminophen + gabapentin combination (AM 251 compared to vehicle pre-treatment; *p* < 0.05) [37]. Pre-treatment with AM 251 significantly attenuated the antinociceptive effect of the acetaminophen + morphine combination with morphine however, a significant residual antinociceptive effect remained (AM 251 compared to vehicle pre-treatment; *p* < 0.05) [37]. Similarly, pre-treatment with AM 630 partially decreased the antinociceptive effect of acetaminophen with morphine, leaving a residual antinociceptive effect (AM 630 compared to vehicle pre-treatment; *p* < 0.05) [37]. Although the acetaminophen + tramadol combination did not produce statistically significant synergy, Hama et al. [37]. found pre-treatment with AM 251 but not AM 630 significantly attenuated the effect of the combination (AM 251 compared to vehicle pre-treatment; *p* < 0.05) [37]. Pre-treatment with either AM 251 or AM 630 before vehicle treatment did not significantly affect withdrawal thresholds [37].

#### Hind paw withdrawal to thermal stimulus

Three studies assessed hind paw withdrawal to thermal stimulus [30, 31, 46]. One study treated rats with WIN 55,212-2 alone or in combination with the CB1 or CB2 receptor antagonists AM 251 and AM 630 [30]. One study involved treatment with AM 281 and AM630 alone and in combination. One study treated mice with CBD [46].

Ahmed et al. found that WIN 55,212-2 treatment after SCI (0.2 mg/kg and 2.0 mg/kg) increased withdrawal thresholds from a thermal noxious stimulus, measured on post-injury day 42, in a dose-dependent manner (WIN 55,212-2 0.2 mg/kg increased hindpaw withdrawal latency from 9.5 ± 0.4 s to 11.1 ± 0.5 s on day 42 post injury; *p* < 0.05. WIN 55,212-2 2 mg/kg increased hindpaw withdrawal latency from = 8.4 ± 0.4 s to 10.7 ± 0.4 s on day 42 post injury; *p* < 0.0001) [30]. Pre-treatment with AM 630 (3 mg/kg) but not AM 251 (3 mg/kg) was found to significantly decrease the anti-hyperalgesic effect of subsequent WIN 55,212-2 i.p. injection (After AM 630 pre-treatment 9.9 ± 0.6 s, after WIN 55–212,2 post-treatment 8.6 ± 1.0 s; *p* > 0.05. After-AM 251 pre-treatment 9.7 ± 0.6 s, after WIN 55–212,2 post-treatment 12.0 ± 0.7 s; *p* < 0.0001) [30].

Arevalo-Martin et al. reported no notable difference in hind paw withdrawal from hot or cold plates when rats were treated with AM 281 (3 mg/kg) and/or AM 630 (3 mg/kg) [31]. A transient increase in hind paw withdrawal time from a cold plate was observed in rats treated with AM 281 or AM 630 at day 60 compared to vehicle-treated rats (*p* < 0.05) but this was not maintained at day 90 [31].

Li et al. found CBD treatment to be associated with a reduction in thermal sensitivity following thoracic contusion injury [46].

### What is the impact of cannabinoids on anxiolysis?

Hong et al. used the elevated plus-maze test to investigate the anxiolytic effects of ACEA post-SCI. No anxiolytic effect of ACEA (3 mg/kg/day) was observed [44].

## Discussion

The aim of this systematic review was to evaluate the effect of cannabinoids on neurobehavioral outcomes in preclinical models of SCI. Overall, cannabinoid receptor agonists ACEA, CBD, CP 55,950, JWH-015, PEA, PEA-OXA and WIN 55,212-2 were reported to produce significant improvements across a range of neurobehavioral outcomes assessing locomotion and pain. However, as aforementioned, a lack of adherence to reporting items on the SYRCLE checklist mean that accurately assessing risk of bias is challenging, and therefore the overall risk of bias and validity of the reported outcomes remain unclear. Comparison between studies and drugs are currently limited by heterogeneity in species, strain, age, injury model, dosing, route of administration, and differences in the timing of neurobehavioural assessments.

### What is the proposed mechanism of action?

WIN 55,212-2 was observed to improve BBB locomotor score, hind paw withdrawal to thermal stimulus and von Frey filament test scores when administered i.p., s.c. and i.t. [30, 33, 35, 36, 38, 40]. However, improvement was only noted at one time point (30 min post administration) in one study [38].

The effects of WIN 55,212-2 were noted to be blocked by CB1 R antagonists in 2 two studies [30, 35] and CB2 R antagonists in two studies [33, 40]. The endo-CB system is thought to be modulated in two phases following SCI [11]. An initial, acute phase in the first week of SCI is characterised by increased levels of the CB1 receptor-specific endo-CB AEA and high levels of CB1 receptor expression on neurons and oligodendrocytes [11]. This acute phase is considered important for neuronal survival [11]. Two to three weeks after SCI, in the chronic phase post-SCI, levels of the non-specific CB receptor endo-CB agonist 2-AG and CB2 receptors in macrophages and astrocyte like cells increase [11]. Increased IL-10 release from CB2 receptor-expressing macrophages has been proposed as a mechanism for CB2 receptor-mediated analgesia post-SCI [30]. Thus, the effects of WIN 55,212-2 following SCI may be mediated by its actions on both the CB1 and CB2 receptors.

Following spinal cord ischaemia, Huo et al. showed WIN 55,212-2 improved motor function, as measured by the 14-point MDI score, reduced apoptosis and improved survival of neurons [40]. Mechanistically, both Huo et al. and Su et al. showed WIN 55,212-2 treatment blocked nuclear translocation of GAPDH, formation of GAPDH/Siah1complexes and iNOS expression in the spinal cord after spinal cord ischaemia or traumatic SCI [33, 40]. GAPDH/Siah1 activity is correlated with apoptosis after ischaemic injury, thus it is proposed that WIN 55,212-2 improves functional recovery following SCI via inhibition of GAPDH/Siah1 signalling cascades and reduction in iNOS expression [33, 40].

Importantly, Hama et al. found the antinociceptive efficacy of WIN 55,212-2 to be maintained over a twice-daily 7-day treatment regimen, whereas the efficacy of morphine decreased over the same period [36]. Tolerance is a major problem with existing SCI-pain medications, hence the sustained efficacy of WIN 55,212-2 suggests that cannabinoid receptor agonists may be useful in alleviating chronic pain after SCI [36].

Potent anti-inflammatory effects of PEA were demonstrated by Genovese et al. who found that following SCI, PEA reduced the degree of spinal cord damage, neutrophil infiltration, NF-kB activation, IkB-a degradation, nitrotyrosine formation, proinflammatory cytokines production, apoptosis, Bax and Bcl-2 expression and PPAR-a degradation [43]. Paterniti et al. provided further evidence supporting the role of PPARs in the mechanism of action of PEA, finding the anti-inflammatory effects of PEA were antagonised by administration of PPARy and PPARd antagonists, and abolished in PPARa KO mice [47]. Furthermore, another study by Paterniti et al. showed a co-ultramicronised composite of PEA and luteolin restored basal expressions of PPAR*a*, *β*, *δ* and *γ* post SCI [48]. Collectively, these studies suggest a significant anti-inflammatory role of PEA post-SCI.

Impellizzeri et al. found PEA-OXA treatment significantly improved BMS scores and reduced histological alterations post SCI. Mechanistically, PEA-OXA was noted to reduce astrocyte activation and increased neurotrophic factors BDNF, GDNF and NT-3 suggesting PEA-OXA has neuroprotective properties. PEA-OXA, similar to PEA, was observed to have anti-inflammatory effects, reducing degradation of IkB-a (a regulatory protein of NF-kb), and reducing expression of iNOS and COX-2, as well as the release of the pro-inflammatory cytokines TNF-a and IL-1b [45].

ACEA treatment was found to improve both functional and histological outcomes post SCI [44]. Hong et al. identified that ACEA treatment decreased matrix metalloproteinase-9 (MMP-9). MMP-9 is known to be expressed in neurons, reactive astrocytes, infiltrating leucocytes and increased activity results in blood-spinal cord barrier disruption and decreased functional recovery following SCI [49].

Two studies implicated the endo-CB system as having a role in the protective effects of RIPC prior to ischaemia/reperfusion injury [41, 34]. Firstly, RIPC has been observed to increase AEA content in the spinal cord following ischaemia/reperfusion injury [34]. Secondly, both Jing et al. and Su et al. reported cannabinoid receptor antagonists reduced the protective effects of RIPC. Jing et al. found the protective effects of RIPC to be reduced at 4 h post-ischaemia/reperfusion injury by CB1, but not CB2, receptor antagonist pre-treatment [41]. At 24 h post-ischaemia/reperfusion injury pre-treatment with either CB1 or CB2 receptor antagonists abolished the neuroprotective effect of RIPC [41]. This implies involvement of both CB1 and CB2 receptors in the protective effects of RIPC. The findings of Su et al. differed to those of Jing et al.. Su et al. report that the protective effects of RIPC were only reversed by CB1 R antagonists at 24 and 48 h post ischaemia/reperfusion injury, implicating involvement of only the CB1 receptor [34]. Therefore, whilst these findings support the involvement of the endo-CB system in RIPC, it is not clear whether both of the cannabinoid receptors are involved.

### Evaluation of pre-clinical evidence base

A large degree of heterogeneity exists in the spinal cord injury models utilised by the included studies, as highlighted in Fig. 2. Each model induces different injuries with different pathophysiology. This is important when considering the potential translation of pre-clinical experiments into human studies. Each model has specific advantages and limitations (summarised in Table 1), and different types of preclinical SCI model may be required to address specific research questions. For example, transection models are a useful method of exploring neuronal regeneration and degeneration, but the pathophysiology of the injury is different to the contusive injury mechanisms more commonly seen in humans [50]. Contusion models are typically regarded as most representative of acute, traumatic SCI, whereas the more chronic injury produced by some compression models may be more representative of conditions such as degenerative cervical myelopathy [51]. The age of animals used provided another source of heterogeneity. Differences in animal age limit interpretation of the included studies as functional behaviours and regeneration after SCI may differ depending on animal age [52]. It is important that future pre-clinical work carefully considers which aspects of human pathology they aim to mimic through animal models, and choose a pre-clinical model and outcomes measures that appropriately reflect this.

There is also heterogeneity amongst the cannabinoid receptor agonists used in the included studies as seen in Fig. 3. WIN 55,212-2 was the most used compound and was noted to produce improvements in both locomotor and pain outcomes [30, 33, 35, 36, 38, 40]. Within those studies that used WIN, various routes of administration were used including intraperitoneal, subcutaneous, intrathecal and intracerebroventricular routes. Furthermore, dosing protocols varied between one off doses and repeat injections. These differences have important clinical implications as some routes of administration (e.g. subcutaneous injection may be easier than others, e.g. intrathecal injection or intracerebroventricular drug administration). The studies included in this review focus primarily on the pharmacodynamics of cannabinoid receptor agonists. However, before clinical trials can be considered, the pharmacokinetics and toxicity of these compounds must also be investigated. Thus, given that multiple compounds have shown improvements in locomotor function and pain scores, future work will need to determine which of these may be most amenable to human translation. This will require essential work investigating pharmacokinetics and distribution, safety and off-target effects, and logistical considerations such as the stability of compounds and shelf-life for use in the clinical environment. Future pre-clinical studies should aim to reach a consensus on lead compounds, explore suitable, clinically relevant dosing regimens, and determine acceptable clinical trade-offs such as the route and frequency of administration.

In our analysis, multiple items from the SYRCLE checklist were not commented on by all 19 studies, including whether animals were randomly selected for outcome assessment, or whether allocation was adequately concealed, and caregivers or investigators were blinded [29]. Due to this, risk of bias remains unclear for the included studies. This appears to be a common problem in the preclinical cannabinoid evidence base. The IASP Presidential Taskforce on Cannabis and Cannabinoid Analgesia identified similar challenges, including the unclear risk of bias due to lack of reporting of methodological criteria [25]. They posit this may be due to reporting of these terms not previously being required by journals for publication [25]. Future pre-clinical trials should be encouraged to follow the ‘Animal Research: Reporting of In Vivo Experiments’ (ARRIVE) guidelines to improve the quality of evidence generated [53].

### Opportunities for translational clinical trials

The beneficial effects of cannabinoid agonists on outcomes assessing pain and locomotor function in animal models of SCI strengthens the argument that there may be scope for the endo-cannabinoid system to be harnessed in the treatment of motor and pain-related symptoms seen following human SCI. Despite many of the drugs discussed in this systematic review not being licensed for clinical use, there are now well-tolerated cannabinoid agonists used in clinical practice. These drugs may represent opportunities for translational clinical trials, bypassing the lengthy and costly process of licensing novel drugs. Sativex and Epidolex are two such drugs. Sativex, an oromucosal spray containing tetrahydrocannbinol and cannabidiol has been licensed in the United Kingdom since 2010 for the treatment of spasticity and other symptoms of multiple sclerosis [54]. Epidolex, a 99% pure oral CBD extract, was the first of its kind to be licensed by the FDA in June 2018 for the treatment of Lennox–Gastaut syndrome, Dravet syndrome and other severe forms of epilepsy [55]. A further three synthetic cannabis-related drug products have since been FDA approved, namely Marinol (dronabinol), Syndros (dronabinol), and Cesamet (nabilone). Existing clinical data from Sativex and Epidolex found diarrhoea, fatigue, somnolence, vomiting and pyrexia to be common adverse events but otherwise noted the drugs to be well tolerated [54, 56, 57]. This provides some insight into how these drugs might be tolerated if they were to be delivered following SCI as part of human clinical trials.

Hama et al. similarly investigated whether combinations of currently licensed drugs (i.e. drug repurposing) could be used to treat pain after SCI [37]. In particular combinations involving acetaminophen, which is proposed to act in part by blocking cellular uptake of anandamide [58, 59]. Combinations of acetaminophen with gabapentin or morphine displayed synergy which was attenuated using CB receptor antagonists implying involvement of the CB receptor activation [37]. The benefits of using such existing drugs include the avoidance of long periods of drug development and licensing as well as the ability to use lower doses to avoid side effects that may otherwise limit the use of such drugs.

Whilst these drugs represent promising opportunities, animal to human translation can be unpredictable and key research questions remain. Firstly, consensus must be reached regarding the optimal cannabinoid drug, dose, and determine what would constitute a clinically acceptable route of administration (particularly if repeated dosing is required). The current preclinical evidence base remains heterogenous, and it is difficult to reconcile inconsistencies between studies when variables such as the model of SCI, timing of drug administration, outcome assessment tool and timing of outcome assessment differ from study to study. Similar variation has been reported in a systematic review of the literature on the effects of cannabinoids in patients who have suffered SCI [23]. We echo their call for appropriately powered, randomised controlled studies with standardised outcome measures, which conform to Consolidated Standards of Reporting Trials, to increase the amount of good quality evidence on this topic. Secondly, there is currently little pre-clinical or clinical literature exploring the long-term effects of chronic cannabinoid use following SCI [23], and this would be worthy of further study. Of the studies included in this review, none explored the effects of administering cannabinoids beyond 10 weeks following SCI. Cannabinoids have been associated with addiction, cognitive decline, sedation, and psychotic disorders [60]. Given that SCI patients may need to use cannabinoids for a number of years, long-term longitudinal studies monitoring the incidence of such side-effects will be required if cannabinoids are to be considered in the management of these patients.

#### Strengths and limitations

Strengths of this review include the following: (1) the search strategy was extensive, (2) each article was independently evaluated for inclusion by 2 reviewers independently evaluated each for eligibility, (3) risk of bias was assessed using the SYRCLE risk of bias tool and (4) the review used the PRISMA guidelines and checklist to structure the review.

This review presents a qualitative, not quantitative, analysis of the existing literature. Meta-analysis was prevented by the low number of studies included and the high degree of heterogeneity in injury model, drug, dose, route of administration, timing of administration, outcome tools, timing of assessment. The overall quality of individual studies was poor due to small sample sizes and failure to satisfy a number of the components of the SYRCLE checklist. Furthermore, due to the relatively small amount of literature on the topic and reliance on what has been reported there is high risk of publication bias. Linking these pre-clinical results to potential clinical findings is difficult given the large differences between animal models of SCI and the injuries seen in patients. Moreover, important side-effects such as sedation and addiction which may have clinical implications were not evaluated in this systematic review. Additional limitations include the exclusion of studies evaluating histological changes and those studies not in English language. Authors of included studies were not contacted to obtain additional information on unpublished or planned studies.

## Conclusion

The results of these studies demonstrate that modulation of the endo-cannabinoid system has significant benefit for both pain and locomotor function across a range of pre-clinical models of acute spinal cord injury. Due to low adherence of reporting items on the SYRCLE checklist, the risk of bias and validity of the reported outcomes remains unclear. Meta-analysis could not be conducted due to small sample sizes and large variation in study design. This highlights the need for additional high-quality preclinical studies with consistent methodology to evaluate the efficacy of cannabinoids in treating SCI. Furthermore, before cannabinoids can be considered in the long-term management of SCI, extensive longitudinal studies are required to monitor the incidence of long-term side effects such as addiction and cognitive decline.

## Supplementary information

Supplementary Material

## Data Availability

Data availability is not applicable to this article as no datasets were generated or analysed during the current study.
