# Supplementary material for: Systematic review of the impact of cannabinoids on neurobehavioral outcomes in preclinical models of traumatic and nontraumatic spinal cord injury
Source: Spinal Cord. 2021 Aug 14;59(12):1221–39. doi: 10.1038/s41393-021-00680-y (PMC8629762; doi:10.1038/s41393-021-00680-y)
Supplement: Supplementary file 1 — Supplementary Material [file 41393_2021_680_MOESM1_ESM.docx]

Systematic Review of the Impact of Cannabinoids on Neurobehavioral Outcomes in Preclinical Models of Traumatic and Nontraumatic Spinal Cord Injury

Supplementary Data

Table of Contents

[Supplementary File 1 – Search Strategy 2](#_Toc76983020)

[Supplementary File 2 – Inclusion and exclusion criteria 3](#_Toc76983021)

[Supplementary File 3 – Reasons for full-text exclusion 4](#_Toc76983022)

[Supplementary File 4 – Summary of drugs acting on the cannabinoid system used in included studies 6](#_Toc76983023)

[Supplementary File 5 – Summary of included studies 7](#_Toc76983024)

[Supplementary File 6 – Risk of bias assessment 20](#_Toc76983025)

[Supplementary File 7 – Synthesis Without Meta-analysis (SWiM) reporting items checklist 21](#_Toc76983026)

# Supplementary File 1 – Search Strategy

**Database: Ovid MEDLINE(R) and Epub Ahead of Print, In-Process & Other Non-**

**Indexed Citations, Daily and Versions(R) <1946 to December 14, 2020>**

Search Strategy:

-------------------------------------------------------------------

1 (Cannabinoid* or TETRAHYDROCANNABINOL or Dronabinol or

THC).mp. or exp *cannabinoids/ (30683)

2 exp Brain Injuries/ (70534)

3 exp Nervous System Diseases/ (2546223)

4 exp Nerve Degeneration/ (25175)

5 exp Spinal Diseases/ (125439)

6 neurotrauma*.mp. (2133)

7 ((neuro* or brain* or nerve*) adj4 (trauma* or demyelinat* or

remyelinat*)).mp. (61069)

8 ((neuro* or brain* or nerve*) adj4 injur*).mp. (166372)

9 concussion*.mp. (13475)

10 or/2-9 (2710876)

11 1 and 10 (3855)

*********************************************

**Database: Embase <1974 to 2020 December 14>**

Search strategy

-------------------------------------------------------------------

1 (Cannabinoid* or TETRAHYDROCANNABINOL or Dronabinol or

THC).ti,ab. or exp *cannabinoids/ (52519)

2 exp *Brain Injuries/ (95986)

3 exp *Nervous System Diseases/ (2222540)

4 exp *Nerve Degeneration/ (17818)

5 exp *Spinal Diseases/ (139176)

6 neurotrauma*.ti,ab. (2878)

7 ((neuro* or brain* or nerve*) adj4 (trauma* or demyelinat* or

remyelinat*)).ti,ab. (84036)

8 ((neuro* or brain* or nerve*) adj4 injur*).ti,ab. (178077)

9 concussion*.ti,ab. (11605)

10 or/2-9 (2419876)

11 1 and 10 (4859)

*********************************************

**Total Search Results 8714**

**With Duplicates Removed 6652**

Search executed 14/12/20. Number of citations for each search term shown in brackets.

# Supplementary File 2 – Inclusion and exclusion criteria

All inclusion criteria had to be met for a study to be included. Studies involving any of the exclusion criteria were excluded.

|  | **Inclusion Criteria** | **Exclusion Criteria** |
| --- | --- | --- |
| **Population** | - Any animal model | - Humans |
| **Injury models** | - Traumatic spinal cord injury - Degenerative cervical myelopathy - Spinal cord ischaemia | - Non-spinal pathologies e.g.   - Root evulsion injuries   - Peripheral nerve injuries (e.g. sciatic nerve)   - Traumatic brain injury   - Epilepsy   - Parkinson’s disease   - Amyotrophic lateral sclerosis   - Transverse myelitis |
| **Intervention** | - Drug acting at cannabinoid receptors (either agonists or antagonists) delivered: - intravenously - intraperitoneally - intrathecally - intracerebroventricularly | - N/A |
| **Comparison** | - Control (vehicle injection) | - Non-drug treatments |
| **Outcomes** | - Neurobehavioral outcomes   - Defined as outcomes of motor and/or sensory function, including pain, using tools such as BBB score for locomotor function and hind foot withdrawal thresholds for thermal hyperalgesia | - Autonomic function or physiological parameters e.g.   - Respiratory function   - Heart rate   - Temperature - In vitro assessment |

# Supplementary File 3 – Reasons for full-text exclusion

| **Author (year)** | **Reason for exclusion** |
| --- | --- |
| Adhikary et al. (2010) | Abstract only - Full text not found |
| Adhikary et al. (2010) | Duplicate |
| Adhikary et al. (2011) | Conference abstract |
| Arevalo-Martin et al. (2012) | Did not evaluate outcomes of interest |
| Baty et al. (2008) | Abstract only - Full text not found |
| Berger et al. (2014) | No spinal cord injury model used |
| Clarke et al. (2001) | Did not evaluate outcomes of interest |
| Esposito and Cuzzocrea et al. (2013) | Literature review |
| Grenald et al. (2018) | Abstract only - Full text not found |
| Heller et al. (2009) | Abstract only - Full text not found |
| Jenkins et al. (2004) | Did not evaluate outcomes of interest |
| Kappel da Silva. (2018) | No spinal cord injury model used |
| Kazantzis et al. (2016) | No spinal cord injury model used |
| Krowicki et al. (1999) | Did not evaluate outcomes of interest |
| LaBuda et al. (2004) | No spinal cord injury model used |
| Li et al (2017) | Did not evaluate outcomes of interest |
| Mohaimany et al. (2017) | Abstract only - Full text not found |
| Mukhopanhyay et al. (2011) | Abstract only - Full text not found |
| Paterniti et al. (2013) | Duplicate |
| Resnick et al. (2010) | Conference abstract |
| Silveira et al. (2014) | No spinal cord injury model used |
| Smith and Martine et al. (1992) | Did not evaluate outcomes of interest |
| Turkanis et al. (1983) | Did not evaluate outcomes of interest |

# Supplementary File 4 – Summary of drugs acting on the cannabinoid system used in included studies

| **Action on Receptor** | **Name** | **Cannabinoid Receptor Selectivity** |
| --- | --- | --- |
| Agonist | Cannabidiol (CBD) | CB_1_ and CB_2_ |
| Agonist | CP 55,940 | CB_1_ and CB_2_ |
| Agonist | JWH-015 | CB_1_ and CB_2_ |
| Agonist | N-(2-chloroethyl)-5Z, 8Z, 11Z, 14Z-eicosatetraenamide (ACEA) | CB_1_ and CB_2_ |
| Agonist | Palmitoylethanolamide (PEA) | CB_1_ and CB_2_ |
| Agonist | Oxazoline of Palmitoylethanolamide (PEA-OXA) | CB_1_ and CB_2_ |
| Agonist | WIN 55,212-2 (WIN) | CB_1_ and CB_2_ |
| Inverse agonist | Rimonabant | CB_1_ |
| Inverse agonist | Hemopressin | CB_1_ |
| Antagonist | AM 251 | CB_1_ |
| Antagonist | AM 281 | CB_1_ |
| Antagonist | AM 630 | CB_2_ |
| Antagonist | SR 144528 (SR 2) | CB_2_ |
| Other | Acetaminophen (APAP) | N/A |
| Other | Naloxone | N/A |

# Supplementary File 5 – Summary of included studies

| **Author (Year)** | **Sample features** | **Injury model** | **Intervention** | **Outcomes Assessed** | **Time of Assessment** | **Statistical Analysis** | **Main Conclusions** |
| --- | --- | --- | --- | --- | --- | --- | --- |
| Ahmed et al (2010) | n = 91-99 (Unclear)  Male Sprague-Dawley rats  Age: Adult  Weight: 250-300 g  Level: T9 | Traumatic SCI  Contusive injury using 10 g rod dropped from height of 12.5 mm | - Sham, operated by laminectomy (n = 14-18)* - Saline vehicle i.p. (n = 7-9)* - DMSO vehicle i.p. (n = 19) - Low-dose WIN 55,212-2 0.2 mg/kg i.p. (n = 14) - High-dose WIN 55,212-2 2.0 mg/kg i.p. (n = 13) - AM 251 3 mg/kg s.c. followed by WIN 55,212-2 2.0 mg/kg i.p. 45 mins after (n = 12) - AM 630 1 mg/kg s.c. followed by WIN 55,212-2 2.0 mg/kg i.p. 45 min after (n = 7-9)*   * indicates unclear sample size. | - Hind paw withdrawal to a noxious thermal stimulus. | - Pre SCI - Post SCI (days 21, 28, 35, 42). - Before drug administration, 45 min after AM 251 or AM 630 administration and 45 min after WIN 55,212-2 administration. | - Two-way ANOVA - Student-Newman-Keuls test for post hoc comparisons | - WIN 55,212-2 (WIN) i.p. significantly ameliorated thermal hyperalgesia in a dose dependent manner. (Low dose WIN 0.2 mg/kg increased hindpaw withdrawal latency from 9.5±0.4 s to 11.1±0.5 s on day 42 post injury; p<0.05. High dose WIN 2 mg/kg increased hindpaw withdrawal latency from 8.4±0.4 s to 10.7±0.4 s on day 42 post injury; p<0.0001.) - AM 251 pre-treatment did not significantly decrease the antihyperalgesic effect of WIN. (Post-AM 251 9.7±0.6 s, post-WIN 12.0±0.7 s; p<0.0001) - AM 630 pre-treatment significantly decreased the antihyperalgesic effect of WIN suggesting a role for the CB2 receptor in modulating SCI-induced thermal hyperalgesia. (Post-AM 630 9.9±0.6 s, post WIN 8.6±1.0 s; p>0.05). |
| Arevalo-Martin et al (2012) | n = 35  Male Wistar rats  Age: 12 weeks  Weight: 295-315 g  Level: T8 | Traumatic SCI  Contusive injury using 200Kdyn for 5 seconds | - Intact, no surgery (n=4) - Vehicle, PBS with 1% BSA i.p. (n=10) - AM 281 3 mg/kg i.p. (n = 6) - AM 630 3 mg/kg i.p. (n = 5) - AM 281 3 mg/kg i.p and AM 630 3 mg/kg i.p (n = 10)   All administered 20 minutes after SCI. | - BBB Locomotor Scale - Hind paw withdrawal from hot and cold plates. - von Frey filament test | - BBB Locomotor Scale: day 7, 14, 30, 60, 90 post SCI. - Hind paw withdrawal to a noxious thermal stimulus: tested twice with a 30 minutes interval, on day 60 and 90 post SCI. | - Kruskal-Wallis test - Non-parametric Mann-Witney test - One-way ANOVA - Bonferroni post-test | BBB Locomotor Scale   - Cannabinoid antagonist-treated rats did not achieve the recovery observed in vehicle treated rats. BBB scores of cannabinoid antagonist-treated rats reached a plateau on day 30 whereas vehicle-treated rats continued to improve until day 60. - Day 7 and 14: No difference observed between vehicle and antagonist-treated rats. - Day 30: Significantly lower BBB values observed in rats treated with AM 630 (p<0.05) and AM 281 + AM 630 (p<0.05) compared to vehicle treated rats. - Day 60: Significantly lower BBB values observed in rats treated with AM 281 (p<0.05), AM 630 (p<0.05) and AM 281 + AM 630 (p<0.001) compared to those of vehicle treated rats. - Day 90: Significantly lower BBB values observed in rats treated with AM 281 (p<0.05), AM 630 (p<0.05) and AM 281 + AM 630 (p<0.05) compared to those of vehicle treated rats.   Von Frey Filament Test (data not shown in paper)   - No difference observed between vehicle- or antagonist-treated rats   Hind paw withdrawal to a noxious thermal stimulus.   - AM 281 and/or AM 630 did not produce notable changes in hot and cold hypersensitivity following SCI compared to rats with SCI only.   Only a transient increase in hind paw withdrawal time in the cold plate was observed in SCI + AM281 and SCI + AM630-treated rats at day 60 compared to SCI treated rats but this was not maintained at day 90. Day 60: SCI + AM281 vs SCI p<0.05. Day 60: SCI + AM630 vs SCI p<0.05. |
| Genovese et al (2008) | n = 30  Male CD1 mice  Age: Adult  Weight: 25-30g  Level: T5-8 | Traumatic SCI  Extradural compression of spinal cord for 1 min using aneurysm clip (24 g) | - Saline vehicle i.p. 30 min before, 1 h after and 6 h after SCI (n = 10) - PEA pre-treatment group: 10 mg/kg i.p. 30 min before SCI, 1h and 6 h after SCI, and daily until day 9 after SCI (n = 10) - PEA post-treatment group: 10 mg/kg i.p. 6 h and 12 h after SCI, and daily until day 9 after SCI (n = 10) | - Basso, Beattie and Bresnahan locomotor score | - Once a day for 10 days after SCI | - Mann-Whitney test | - Palmitoylethanolamide (PEA) pre- or post-treatment significantly ameliorated the functional deficits induced by SCI (p < 0.05) - No significant different was found in the ability to reduce spinal cord injury by PEA administered as pre- or post- treatment. |
| Hama et al (2007) | n = 48  Male Sprague-Dawley rats  Age: Not specified  Weight: 125-150 g at time of SCI  Level: T6-8 | Traumatic SCI  Compression of spinal cord with microaneurysm clip (20 g) for 1 minute | Agonist Experiment (n = 24)   - Vehicle s.c. (n = 6) - WIN 55,212-2 0.3, 1, 3 mg/kg (n = 6/ group)   Antagonist Experiments (n = 24 – same rats used for both antagonists in pseudo-Latin square design)   - Vehicle s.c. + Vehicle s.c. (n = 6/ experiment) - Vehicle s.c. + WIN 55,212-2 3 mg/kg s.c. (n = 6/ experiment) - AM 251 s.c 3 mg/kg or AM 630 3 mg/kg + vehicle s.c. (n = 6/ antagonist) - AM251 s.c. 3mg/kg or AM 630 3 mg/kg + WIN 55,212-2 s.c. (3mg/kg) (n = 6/ antagonist)   All drugs administered 4-5 weeks after SCI. In antagonist experiments, WIN 55,212-2 or vehicle was injected 30 minutes after the first injection of antagonist or vehicle. | - von Frey filament test | - Agonist Experiment: Baseline taken after SCI. Once every 30 min for 120 min following injection. - Antagonist Experiment: Baseline taken after SCI before. 30 minutes after vehicle/AM 251/ AM 630 injection. 30 minutes after vehicle/WIN 55,212-2 injection. | - Repeated-measure two-way ANOVA - Student-Newman-Keuls test for post hoc comparisons | Agonist experiment   - WIN 1 mg/kg and 3 mg/kg s.c. produced dose dependent increases in withdrawal thresholds at 30 min and throughout remaining observation period, indicating a reversal in mechanical hypersensitivity (p<0.05 vs vehicle at all time points). - WIN 0.3 mg/kg s.c. and vehicle did not significantly alter withdrawal thresholds. - 50% analgesic dose of WIN 30 min after injection was 0.7 (95% C.L. = 0.5-1.0) mg/kg.   Antagonist experiment   - AM 251 pre-treatment blocked the antinociceptive effect of WIN. 30min after injection of WIN the withdrawal threshold of the AM 251/WIN group (2.1±0.4 g) was significantly less than that of the vehicle/WIN group (15± 0.0 g; p<0.05) and was not different from the threshold of the vehicle/vehicle group (2.4± 0.3 g; p>0.05).   AM 630 pre-treatment did not significantly alter the antinociceptive effect of WIN. 30 min after injection of WIN, the withdrawal threshold of the AM 630/WIN group (14.4 ± 0.6 g) was not significantly different from the withdrawal threshold of the vehicle/WIN group (15 g; p > 0.05), but was significantly different from the threshold of the vehicle/vehicle group (2.1 ± 0.2 g; p < 0.05) and AM 630/vehicle group (2.9 ± 0.4 g; p < 0.05). |
| Hama et al (2009) | n = 23  Male Sprague-Dawley rats  Age: Not specified  Weight: 100-125g at time of SCI  Level: T6-7 | Traumatic SCI  Compression of spinal cord with microvascular clip (20 g) for 1 minute. | - Vehicle s.c. twice daily (8 am and 5 pm for 7 days, 4 weeks after SCI (n = 5-6)* - WIN 55,212-2 0.3, 1, 3 mg/kg s.c. twice daily (8 am and 5 pm) for 7 days, 4 weeks after SCI (n = 5-6/group)*   * indicates unclear sample sizes | - von Frey filament test | - Before SCI - Before and 30 min after 8am injection. | - Two-way ANOVA - Student-Newman-Keuls test | - WIN s.c. produced a dose-dependent increase in withdrawal thresholds 30 min post-injection on Day 1 (p < 0.05) - The antinociceptive effect of WIN was maintained for the duration of the 7 day experimental period. - The 50% antinociceptive (A50) dose (95% confidence interval) on day 1 was not significantly different from day 7: 0.9 (0.6–1.2) mg/kg vs 0.8 (0.6–1.2) mg/kg. - The percent maximum possible effects (MPE) of 3 mg/kg s.c. WIN did not significantly change from day 1 (99% ± 1%) to day 7 (88% ± 9%). |
| Hama et al (2010) | n = 452-488 (Experiment 1, n = 56. Experiment 2, n = 396-432, with n = 6-8 per treatment group)  Male Sprague-Dawley rats  Age: Not specified  Weight: 140-160g at time of surgery  Level: T6-7 | Traumatic SCI  Compression of spinal cord with microvascular clip (20 g) for 1 minute. | Experiment 1:   - Acetaminophen (APAP) (n = 6-8/group)* - Gabapentin (n = 6-8/group)* - Memantine (n = 6-8/group)* - Morphine (n = 6-8/group)* - Tramadol (n = 6-8/group)* - APAP + gabapentin (100+26, 50+13, 25+6.5, 12.5+3.25 mg/kg) (n = 6-8/group) - APAP + memantine (100+8, 50+4, 25+2 mg/kg) (n = 6-8/group) - APAP + morphine (100+1, 30+0.3, 10+0.1 mg/kg) (n = 6-8/group) - APAP + tramadol (100+20, 50+10, 25+5, 12.5+2.5 mg/kg) (n = 6-8/group)   Experiment 2:  Pre-treatment followed by post-treatment 30 min after   - Vehicle/ Vehicle (n = 7/group) - Vehicle/ APAP + active drug (n = 7/group) - Antagonist/ Vehicle (n = 7/group) - Antagonist/ APAP + active drug (n = 7/group)   This was done for each active drug:   - Acetaminophen 3 ml/kg i.p. - Gabapentin 1 ml/kg s.c. - Memantine HCl 1 ml/kg s.c. - Memantine sulphate 1 ml/kg s.c. - Tramadol HCl 1 ml/kg s.c.   With the following antagonists:   - AM251 3 mg/kg s.c. - AM630 1 mg/kg s.c. - Naloxone 5 mg/kg s.c.   * indicates unclear sample sizes | - von Frey filament tests | - Baseline 4 weeks after spinal cord compression. - Experiment 1: Every 30 min up to 120 min post-injection. - Experiment 2: 60 or 90 min after the second injection depending on the particular combination. | - Two-way ANOVA - Student-Newman-Keuls test | Acetaminophen (APAP) alone:   - Highest injectable dose of APAP was 100 mg/kg. At 60 min and 90 min post-injection, the percent maximum possible effects (MPEs) were 27 ± 8% and 33 ± 11%, respectively (p > 0.05 vs vehicle).   APAP + Gabapentin:   - Combination of the two drugs was synergistic. There was a 2.6-fold leftward shift of the APAP + gabapentin dose-response curve compared to that of gabapentin alone and the experimentally determined A_50_ of the combination was significantly less than the theoretical A_50_ (p < 0.05). - Pre-treatment with AM251 significantly attenuated the antinociceptive effect of APAP +gabapentin (p < 0.05 vs. veh/APAP+GP). No significant difference was observed between the AM251/vehicle and AM251/APAP+gabapentin treated groups (p > 0.05). Pre- treatment with AM630 did not significantly attenuate the effect of the APAP+gabapentin combination (p > 0.05 vs. vehicle/APAP+GP).   APAP + Memantine:   - Combination of the two drugs was merely additive. The experimentally determined A_50_ of the combination (at 60 min post-injection) was not significantly different from the theoretical A_50_ (p > 0.05). No significant shift in potency was observed between the combination and memantine alone.   APAP + Morphine:   - Combination of the two drugs was synergistic. The experimentally determined A_50_ of the combination was significantly less than the theoretical A_50_ (p < 0.05). There was a 3.4-fold leftward shift in potency of APAP+morphine compared to morphine alone. - Pre-treatment with AM251 significantly attenuated the effect of APAP+morphine (p < 0.05 vs. veh/APAP+Mor), however a significant “residual” antinociception was still observed in AM251-pretreated rats such that the mean withdrawal threshold was still higher than that of vehicle post-treated rats (p < 0.05 vs. AM251/veh). Pretreatment with AM630 partially decreased the antinociceptive effect of APAP+morphine (p < 0.05 vs. veh/APAP+Mor). Similar to pre-treatment with AM251, a “residual” antinociception in the AM630/APAP +morphine group was observed (p < 0.05 vs. AM630/veh).   APAP + Tramadol:   - Combination of the two drugs was merely additive. A 2-fold leftward shift in potency of APAP+tramadol compared to tramadol alone was observed, however the experimentally determined A_50_ of the combination (at 90 min post-injection) was not statistically significant from the experimentally determined A_50_ (p > 0.05). - Pre-treatment with AM 251 significantly attenuated the effect of APAP+tramadol (p < 0.05 vs. veh/APAP+Tram). - Pre-treatment with AM 630 did not affect APAP +tramadol antinociception (p > 0.05). - Pre-treatment with naloxone significantly attenuated the effect of the APAP+tramadol combination (p < 0.05 vs. veh/APAP +Tram).   Antagonists   - Pre-treatment with either AM 251 3 mg/kg s.c., AM630 1 mg/kg s.c., naloxone 5 mg/kg s.c., or vehicle did not significantly affect withdrawal thresholds of SCI rats post-treated with vehicle (p > 0.05). - AM 251 pre-treatment 30 min prior to morphine 3 mg/kg, gabapentin 100 mg/ kg, or tramadol 100 mg/kg treatment did not significantly alter of the morphine, gabapentin or tramadol when administered alone (p > 0.05; data not shown). - AM 630 pre-treatment 30 min prior to morphine3 mg/kg treatment did not significantly change the efficacy of morphine when administered alone (p > 0.05; data not shown). |
| Hama et al 2011 | n = 198-206 (Unclear)  Male Sprague-Dawley rats  Age: Not specified  Weight: 100-150g at time of SCI.  Level: T6-7 | Traumatic SCI  Compression of spinal cord with microvascular clip (20 g) for 1 minute. | Experiment 1: Antinociceptive effects of drugs (n = 94-102)   - WIN 55,212-2 (vehicle, 5.7, 19.1, 57.4nmol) i.t. (n = 6-8/group)* - WIN 55,212-2 (vehicle, 1.9, 5.7, 19.1 nmol) i.c.v. (n = 7/group) - Hemopressin (vehicle, 0.09, 0.9, 9.2 nmol) i.t. (n = 6/group) - Rimonabant (vehicle, 6.5, 64.7 nmol) i.t. (n = 6/ group)   Experiment 2: Effects of pre-treatments on antinociceptive effects of WIN 55,212-2 (n=104)   - Vehicle pre-treatment / Vehicle post (n = 6/7) - Vehicle pre-treatment / WIN 55,212-2 (n = 6/7) - Antagonist pre-treatment / Vehicle (n = 6/7) - Antagonist pre-treatment / WIN 55,212-2 (n = 6/7)   This was done 4 times:   1. Hemopressin 2.8 nmol i.t. or vehicle i.t. pretreatment 30 min before WIN 55,212-2 57.4 nmol i.t. or vehicle i.t. injection (n=7 per group) 2. Hemopressin 2.8 nmol or vehicle pretreatment coinjected i.c.v. with WIN 55,212-2 19.1 nmol or vehicle . (n=7 per group) 3. Rimonabant 64.7 nmol i.t. or vehicle i.t. pretreatment 30 min before WIN 55,212-2 57.4 nmol i.t. or vehicle i.t. injection (n = 6/group) 4. Rimonabant 3mg/kg s.c. or vehicle i.c.v. pre-treatment 30 min before WIN 55,212-2 19.1 nmol i.c.v. or vehicle i.c.v. injection (n = 6 per group)   * indicates unclear sample size | - von Frey filament test | - Experiment 1: Prior to drug administration and every 30 min up to 2 h post injection. - Experiment 2: Prior to, and 30 min after vehicle or WIN 55,212-2 treatment. | - Repeated-measure two-way ANOVA - Two-way ANOVA. - Student–Newman–Keuls test for post hoc comparisons. | Effect of centrally administered CB receptor ligands in SCI rats   - WIN 55,212-2 57.4 nmol i.t. significantly increased withdrawal thresholds 30 min injection, this effect lasted at least 120 min post-injection (p<0.05 vs. vehicle). At 30 min post-injection, the percent maximum possible effect (MPE) was 42.5±12.0%. No significant effects on withdrawal thresholds were observed with lower doses of WIN 55,212-2 or vehicle. - WIN 55,212-2 i.c.v produced a dose-dependent antinociceptive effect 30 min following injection (p<0.05 vs. vehicle). At 30 min, the 50% antinociceptive dose (A50) (95% confidence limits) was 5.0 (2.7-9.3) μg. No significant antinociceptive effect was observed at any other time post-injection. Vehicle injection did not significantly alter withdrawal thresholds. - Hemopressin i.t. and rimonabant i.t. did not significantly alter hind paw withdrawal thresholds.   Effects of pre-treatments on the antinociceptive effect of WIN 55,212-2   - Hemopressin did not alter the antinociceptive effect of WIN 55,212-2, either as an i.t. pre-treatment or as an i.c.v. cotreatment. - Rimonabant i.t., but not vehicle, pre-treatment blocked the antinociceptive effect of WIN 55,212-2 i.t. (p<0.05, Rimonabant/WIN 55,212-2 vs. Vehicle/WIN 55,212-2).   Rimonabant s.c., but not vehicle, blocked the antinociceptive effect of i.c.v. WIN 55,212-2 (p<0.05, Rimonabant/WIN 55,212-2 vs. Vehicle/WIN 55,212-2). |
| Hama et al (2014) | n = 120  Male Sprague-Dawley rats  Age: Not specified  Weight: 100-130g at time of SCI  Level: T6-7 | Traumatic SCI  Compression of spinal cord with microvascular clip (20 g) for 1 minute. | Experiment 1: Acute dose of CP 55,940 (n = 30)   - Vehicle s.c. once (n=6) - CP 55,940 0.01, 0.03, 0.1, 0.3 mg/kg s.c. once (n = 6/group)   Experiment 2: Repeat drug treatment - injected twice daily (8 am and 5pm) for 7 days except amitriptyline which was injected once daily (n = 60)   - CP 55,940 0.3mg/kg s.c. or vehicle s.c. (n = 6/group) - Amitriptyline s.c. 30mg/kg or vehicle s.c. (n = 8/group) - Gabapentin 25mg/kg s.c. or vehicle s.c. (n = 8/group) - Carbamazepine 30mg/kg s.c. or vehicle s.c. (n = 8/group)   Experiment 3: Pre-treatment with CB receptor antagonists on the antinociceptive effect of CP 55,940. (n = 36)   - Vehicle s.c. pre-treatment + Vehicle s.c. (n = 6) - Vehicle s.c. pre-treatment + CP 55,940 0.3mg/kg s.c. (n = 6) - Rimonabant 3 mg/kg s.c. pre-treatment + Vehicle s.c. (n = 6) - Rimonabant 3 mg/kg s.c. pre-treatment + CP 55,940 0.3mg/kg s.c. (n = 6) - SR 144528 1 mg/kg s.c. pre-treatment + Vehicle s.c. (n = 6) - SR 144528 1 mg/kg s.c. pre-treatment + CP 55,940 0.3mg/kg s.c. (n = 6)   Pre-treatment administered 30 min before treatment with vehicle or CP 55,940. | - von Frey filament test | - Experiment 1: Before injection, every 30 min up to 120 min post injection. - Experiment 2: After 8 am injection for 7 days. - Experiment 3: Before pre-treatment and 30 and 60 min after treatment. | - Two-way ANOVA - Student-Newman-Keuls for post hoc comparisons. | Experiment 1: Antinociceptive effect of CP 55,940   - CP 55,940 significantly increased withdrawal threshold in a dose-dependent manner (P < 0.05 vs. vehicle). - Maximum efficacy was rapidly obtained following injection with the highest dose (0.3 mg/kg). - Maximum efficacy with lower doses (0.03 and 0.1 mg/kg) was not observed until 60-90 min following injection. - The A50 (dose producing a 50% antinociceptive effect) (95% CL) at 60 and 90 min post injection was 0.05 (0.03–0.07) mg/kg and 0.04 (0.03–0.05) mg/kg, respectively.   Experiment 2: Efficacy following repeated dosing   - The antinociceptive effect of CP 55,940 on threshold values was maintained, at full efficacy, with twice daily dosing over a 7-day observation period.   Experiment 3: Pre-treatment with CB1 and CB2 selective receptor antagonists  Pre-treatment with rimonabant, a CB1 receptor antagonist, blocked the effect of CP 55,940 (P < 0.05, rimonabant pre-treatment vs. vehicle pre-treatment) whereas pre-treatment with SR 144528, a CB2 receptor antagonist, did not block the effect of CP 55,940 (P > 0.05, SR 144528 pre-treatment vs. vehicle pre-treatment) |
| Hong et al (2015) | n = 53  Male C57BL/6J mice  Age: Young adult  Weight: 20-25g  Level: T11 | Traumatic SCI  1.5 mm silicon tube placed into the T11 vertebral canal for 60 min. | Rodent rotarod and 9-point Basso Mouse scale   - Vehicle i.p. for 21 days beginning 1 h post spinal cord compression (n = 13)* - ACEA 3mg/kg/day i.p. for 21 days beginning 1 h post spinal cord compression (n = 12)   Spontaneous open field locomotor activity   - Normal mice, no SCI (n = 5) - Vehicle once (n = 6) - ACEA 3 mg/kg i.p. once (n = 6)   Elevated plus maze test   - Vehicle i.p. (n = 6) - ACEA 3 mg/kg/day i.p. for 2 weeks (n = 6)   * Started with 13 mice however 1 died thus only 12 were observed throughout the full experimental period. | - Rodent rotarod - Basso Mouse scale - Spontaneous open field locomotor activity - Elevated plus maze test (looking at anxiolytic effects) | - Rodent rotarod: 1 day prior to and 1, 2, 7, 14, and 21 days after injury. - Basso Mouse scale for locomotion: 21 days after injury. - Spontaneous open field locomotor activity: 1, 2, 4, 24 hours post treatment. - Elevated plus maze test: after 2 weeks of treatment. | - Repeat measures of ANOVA - One-way ANOVA - Non-parametric Mann Whitney U statistic. | - Hindlimb function partially recovered in both vehicle and ACEA 3mg/kg/day treated mice. - Mice treated with ACEA 3mg/kg/day demonstrated improved rotarod function over the 21-day recovery interval versus the vehicle-treated group (P=0.04). - Both vehicle and ACEA treated mice were able to use the hindlimbs to support body weight by 3 weeks (BMS score >5). - Numerically, a greater number of mice were able to coordinate forelimb, hindlimb and tail function (BMS = 9) in the ACEA treatment group (5 of 12) versus vehicle treatment group (0 of 11). - The ACEA treatment group had better BMS scores at 21 days compared to vehicle (ACEA = 8 ± 3, vehicle = 5 ± 6, p = 0.02). - ACEA did not affect post-injury spontaneous activity or produce anxiolysis measured using elevated plus maze test. |
| Huo et al (2018) | n = 40  Male Sprague-Dawley rats  Age: NS  Weight: 300-350g  Level: Catheter tip was at the level of the subclavian artery. | Spinal cord ischaemia  Catheter occlusion of aorta (catheter tip at the level of the subclavian artery) for 12 min. | - Sham surgery + vehicle i.p. 8 h and 24 h after reperfusion (n = 10) - Vehicle control i.p. 8 h and 24 h after reperfusion (n = 10) - WIN 55,212-2 1 mg/kg i.p. immediately, 8 h and 24 h after reperfusion (n = 10) - WIN 55,212-2 1 mg/kg i.p. immediately, 8 h and 24 h after reperfusion + AM 251 1 mg/kg i.p. 30 min before each injection of WIN 55,212-2 (n = 10) - WIN 55,212-2 1 mg/kg i.p. immediately, 8 h and 24 h after reperfusion + AM 630 1 mg/kg i.p. 30 min before each injection of WIN 55,212-2 (n = 10) | - 14-point motor deficit index (MDI) score | - 48 hours after reperfusion | - Mann-Whitney U test | - WIN 55,212-2 treatment significantly improved motor deficit index (MDI) scores following ischaemic SCI compared to the control group (Control 5 [2], vs WIN 2.5 [1.25]; p < 0.05) - CB2 receptor antagonism reversed the effects of WIN 55,212-2. The MDI score of the WIN + AM630 (CB2 R antagonist) treated group was greater than that of the WIN treated group (p < 0.01), while there was no significant difference between the scores of WIN and WIN + AM251 (CB1 R antagonist) treated groups (WIN, 2.5 [1.25], vs WIN + AM630, 5 [1.25], WIN + AM251 2.5 [1.25]) - Data expressed as ‘median [interquartile range]’ |
| Impellizzeri et al (2017) | n = 60  Male CD1 mice  Age: Adult  Weight: 25-30g  Level: T6-7 | Traumatic SCI  Extradural compression with aneurysm clip (24 g) for 1 min | - SCI + Vehicle i.p. or orally (o.s.) (n = 10) - SCI + PEA-OXA 10 mg/kg i.p. (n = 10) - SCI + PEA-OXA 10 mg/kg o.s. (n = 10) - Sham-operated + Vehicle i.p. or o.s. (n = 10) - Sham + PEA-OXA 10 mg/kg i.p. (n = 10) - Sham + PEA-OXA 10 mg/kg o.s. (n = 10)   PEA-OXA i.p. and o.s. was administered 1h and 6h after SCI. | - Basso Mouse Scale | - Daily for 10 days after SCI. | - Two-way ANOVA - Bonferroni post-tests | - PEA-OXA treatment (10 mg/kg) ameliorated the functional deficits induced by SCI (p < 0.05). - No significant difference was found between intraperitoneal or oral administration of PEA-OXA. |
| Jing et al (2017) | n = 168  Male Sprague-Dawley rats  Age: NS  Weight: 280-320g  Level: N/A | Ischemia/Reperfusion Injury  Cross-clamp applied to aortic arch between the left common carotid artery and left subclavian artery for 14 minutes. | - Sham, no remote ischaemic preconditioning (RIPC) or SCI (n = 36) - Control, SCI but no prior RIPC (n = 36) - RIPC, 3 cycles of RIPC prior to SCI (n = 36) - Vehicle i.p. 15 min prior to RIPC, followed by SCI (n = 36) - AM251 1 mg/kg i.p. 15 min prior to RIPC, followed by SCI (n = 36) - AM630 1 mg/kg i.p. 15 min prior to RIPC, followed by SCI (n = 36) | - Tarlov scoring system | - 4 h and 24 h after reperfusion. | - Kruskal-Wallis - Freidman tests - Dunn’s multiple comparison test. | - RIPC enhanced the recovery of motor function assessed using the Tarlov score (RIPC group vs control group; P < 0.05, at 4 h and 24 h respectively). - At 4 h after reperfusion, the neurological outcome in the AM 251 pre-treatment before RIPC group was significantly worse than the RIPC only group (P < 0.05). The scores in RIPC, AM630, and vehicle groups did not significantly differ. - At 24 h after reperfusion, both AM251 and AM630 pre-treatment statistically abolished the neuroprotective effect of RIPC (AM 251 pre-treatment and AM 630 pre-treatment groups vs RIPC group; P < 0.05 respectively). |
| Kwiatkoski et al (2012) | n = 28  Male Wistar rats  Age: NS  Weight: 280-300g  Level: T10 | Cryogenic SCI  Liquid nitrogen jet applied to spinal cord for 5 seconds | - Naive control, no surgery (n = 4) - Laminectomy, no SCI (n = 8) - Lesion + Vehicle i.p. immediately before, 3 h after, and daily for 6 days after SCI (n = 8) - Lesion + CBD 20 mg/kg i.p. immediately before, 3 h after, and daily for 6 days after SCI (n = 8) | - Basso, Beattie, Bresnahan Locomotor score | - Before surgery and on the 1st, 3^rd^, and 7^th^ day after surgery. | - Two-way ANOVA - Bonferroni post hoc test. | - BBB scores did not differ between the animal groups before the test, all reaching the maximum BBB score of 21. - Day 1 after injury, all lesioned animals had a zero score on the BBB scale (no movement of the hind limbs). - Day 3 after injury, BBB scores of CBD treated animals (median = 2) where greater than vehicle treated animals (median = 0.5); P < 0.05 (Dunn’s test). - Day 7 after injury, CBD treated animals continued to obtain higher BBB scores (median = 7.0, meaning ample movement of the three joints of the hind limbs) compared to vehicle treated animals (median = 4.5, with 4 points meaning discrete movement of the thee joints of the hind limbs); p < 0.05 (Dunn’s test). - Both CBD and vehicle treatment groups achieved improvements in BBB score improvement from the Day 1 to Day 7; p < 0.01 (Friedman test). |
| Latini et al (2014) | n = 40  Male Wistar rats  Age: Adult  Weight: 200-250g  Level: C4 | Traumatic SCI  Lateral cervical spinal cord hemisection (SCH) using iridectomy scissors. | - Control, no SCI (n = 10) - SCH + JWH-015 3 mg/kg i.p. daily for 7 days (n = 10) - SCH + SR2 3 mg/kg i.p. daily for 7 days (n = 10) - SCH + Saline i.p. daily for 7 days (n = 10) | - Beam-walking test - catWalk | - Beam walking: Day 0 (control), 1, 3, 5, 7, 21 and 60 days after surgery. - catWalk: Day 7, 21, 60 | - Mixed-effects linear regression or mixed-effects logistic regression/ generalised estimating equation (GEE) analysis - Turkey-Kramer multiple comparison post-test adjustments | - Beam-walking test: - Day 1 after SCH: Saline, JWH, SR2 treatment groups exhibited uniform, severe motor impairments. - Day 3 onwards: JWH treated animals obtained better beam-walking scores than saline treated animals (p <0.001) and SR2 treated animals (p < 0.0001). - No differences were observed between saline and SR2 treatment groups. - CatWalk: - All SCH groups developed clear deficits in all parameters on day 7. - In subsequent days, all groups generated recovery curves that never reached control values. JWH treatment group CatWalk scores significantly differed from saline and SR2 treated animals at all time points; - No differences were observed between the saline and SR2 treatment groups. |
| Li et al (2018) | n = 25  Female C57BL/6 mice  Age: 7-9 weeks  Weight: 16-21g  Level: T9-T10 | Traumatic SCI  Contusive injury by impactor drop from 3mm to deliver 60 kdyn force to spinal cord. | - Sham, no SCI (n = 10) - SCI + Vehicle i.p. 1 h, 24 h and on day 3 post-surgery, then twice a week for 10 weeks (n = 7) - SCI + Cannabidiol 1.5 mg/kg i.p 1 h, 24 h and on day 3 post-surgery, then twice a week for 10 weeks (n = 8) | - Basso Mouse scale - Hindpaw withdrawal from thermal stimulus - von Frey filaments test | - Basso Mouse Scale: Day 1, 3, 7 and thereafter once weekly for 10 weeks - Hindpaw withdrawal from thermal stimulus: before surgery then week 4, 6, and 10 after surgery. - Von Frey filaments test: before surgery then week 2, 4, 6, 8, 10 after surgery. | - One-way ANOVA - Newman-Keuls post-hoc analysis | Basso Mouse Score   - CBD treatment following SCI had no significant effect on BMS scores compared to the vehicle treatment.   Thermal Hypersensitivity   - 10 weeks post-SCI, median percent thermal threshold scores compared to baseline were approximately 50% in vehicle treated mice and 70% in CBD treated mice. - Mixed-effects linear regression showed a significant main effect of CBD treatment [F(2, 22) = 20.75, p<0.0001], but no significant main effect of time [F(2, 44) < 1, n.s.] and no significant interaction between CBD treatment and time [F(4, 44) = 1.17, n.s.]. - Tukey’s multiple comparison post-tests showed that at Week 4, both SCI-VEH and SCI-CBD mice were significantly more sensitive compared with SHAM mice, at Week 6 only SCI-VEH mice were significantly different from SHAM mice, and at Week 10 both SCI-VEH and SCI-CBD mice showed significantly more sensitivity compared with SHAM mice. - Analysis using two sensitivity categories (“mild” = ≥66 - 100% baseline thermal threshold response and “moderate to severe” = 0 – 65.9% baseline thermal threshold response): Approximately 70% of vehicle treated mice went developed moderate to severe thermal sensitivity that progressed in severity between 4 and 10 weeks post-SCI whereas only 25% of CBD treated mice developed moderate to severe thermal sensitivity. GEE/mixed-effects logistic regression analysis showed a significant effect of treatment (p=0.01) but no significant effect of time (p=0.85). Post-test analysis showed that vehicle treated mice had a significantly higher proportion of “moderate to severe” than the sham injured mice across time (p<0.01). CBD treated mice were not significantly different from SHAM mice across time.   Mechanical sensitivity   - Mixed linear regression analysis revealed no statistically significant main effect of treatment [F(2,22) = 0.98, p=0.39], time [F(8,175) = 1.03, ns], or interaction of treatment and time [F(16,175) =1.17, ns]. - Analysis of more sensitive and less sensitive mechanical sensitivity values obtained by mice at each time point: - When the more sensitive percent baseline scores for each mouse (left paw vs right paw) were averaged from each animal at each time point, it was observed that vehicle and CBD treated mice displayed higher sensitivity to touch than sham mice. Mixed-effects linear regression analysis revealed a marginally significant main effect of treatment [F(2,22) = 2.42, p=0.11], and no statistically significant main effect of time [F(8,175) = 1.05, ns], and no statistically significant interaction between treatment and time [F(16,175) =1.16, ns]. Tukey-Kramer’s post-tests showed (marginally) significant differences between CBD and sham-injured groups at week 4 (p=0.08) and between vehicle and sham-injured groups at week 4 (p=0.06), week 6 (p=0.03), and week 8 (p=0.02).   When the less sensitive percent baseline scores for each mouse (left paw vs right paw) were averaged, it was observed that vehicle and CBD treated mice displayed less sensitivity to touch than sham mice. Mixed-effects linear regression analysis revealed a statistically significant main effect of treatment [F(2,22) = 3.70, p=0.04], and no statistically significant main effect of time [F(8,175) = 1.32, ns], and no statistically significant interaction between treatment and time [F(16,175) = 0.78, ns]. Tukey-Kramer’s post-tests showed marginally significant differences between SCI-CBD and SHAM groups at week 6 (p=0.09), and marginally significant differences overall among the three groups at week 2 (p=0.03), week 5 (p=0.07), and week 6 (p=0.07). |
| Paterniti et al (2013a) | n = 390  PPAR-$\alpha$KO mice (homozygous for the Ppara^tm1Gonz^ targetted mutation, strain name: 129S4/SvJae-Ppara^tm1Gonz^/J) and litter-mate wildtype controls. Gender not stated.  Age: 6-7 weeks  Weight: 20-27g  Level: T6-7 | Traumatic SCI  Extradural compression with aneurysm clip (24 g) for 1 minute. | - WT mice, sham + vehicle i.p. 1 h, 6h post-surgery (n = 30) - WT mice, sham + PEA 10 mg/kg i.p. 1 h, 6h post-surgery (n = 30) - WT mice, sham + vehicle i.p. 1h, 6h post-surgery + GW9662 1 mg/kg i.p. 30 min prior to vehicle injections (n = 30) - WT mice, SCI + vehicle i.p. 1 h, 6h post-surgery (n = 30) - WT mice, SCI + PEA 10 mg/kg i.p. 1 h, 6h post-surgery (n = 30) - WT mice, SCI + vehicle i.p. 1 h, 6h post SCI + GSK0660 1 mg/kg i.p. 30m and 5h post-SCI (n = 30) - WT mice, SCI + PEA 10 mg/kg i.p. 1h, 6h post-SCI + GSK0660 1 mg/kg i.p. 30 min prior to PEA injections (n = 30) - PPAR-$\alpha$KO mice , sham + vehicle i.p. 1 h, 6h post-surgery (n = 30) - PPAR-$\alpha$KO mice, sham + PEA 10 mg/kg i.p. 1 h, 6h post-surgery (n = 30) - PPAR-$\alpha$KO mice, SCI + vehicle i.p. 1 h, 6h post-SCI (n = 30) - PPAR-$\alpha$KO mice, SCI + PEA 10 mg/kg i.p. 1 h, 6h post-surgery (n = 30) - PPAR-$\alpha$KO mice, SCI + vehicle i.p. 1 h, 6h post-SCI + GW9662 1 mg/kg i.p. 30 min and 5h post-SCI (n = 30) - PPAR-$\alpha$KO mice, SCI + PEA 10 mg/kg i.p. 1h, 6h post-SCI + GW9662 1mg/kg 30 min prior to PEA injections (n = 30) | - Basso Mouse Scale | - Prior to surgery and everyday post injury for 10 days. | - Two-way ANOVA followed by Bonferroni post-hoc analysis - Mann-Whitney test | - Palmitoylethanolamide (PEA) treatment 10 mg/kg caused a significant increase in BMS scores; p < 0.05 - Pre-treatment with GSK0660 (PPAR-d antagonist) or GW9662 (PPAR-y antagonist) abolished PEA-induced improvement of locomotion after SCI - The genetic absence of the PPAR-$\alpha$ receptor in PPAR-$\alpha$KO mice significantly blocked the effect of the PEA treatment. |
| Paterniti et al (2013b) | n = 50  CD1 mice (gender not specified)  Age: NS  Weight: NS  Level: T6-7 | Traumatic SCI  Extradural compression with aneurysm clip (24 g) for 1 minute. | - Sham + vehicle i.p. (n = 10) - SCI + vehicle i.p. (n = 10) - SCI + Palmitoylethanolamide (PEA) 1 mg/kg i.p. 1h and 6h post-SCI (n = 10) - SCI + PEA 0.9 mg/kg i.p. and Luteolin 0.1 mg/kg association 1h and 6h post-SCI (n = 10) - SCI + co-ultramicronized composite of PEA and Luteolin (co-ultraPEALut) 1 mg/kg i.p. 1h and 6 h post-SCI (n=10) | - Basso Mouse Scale | - Daily for 10 days after SCI. | - Mann-Whitney test | - SCI produced significant deficits in movement assessment using the Basso Mouse scale. - Treatment with PEA alone or Lut alone did not ameliorate the functional deficits induced by SCI. - The PEA + Lut association, given as a single treatment combination did not restore motor function. - Treatment with Co-ultraPEALut 1 mg/kg significantly reduced the motor disturbance after SCI compared vehicle treatment; p < 0.01. |
| Su et al (2017) | n = 50  Male Sprague-Dawley rats  Age: NS  Weight: 260-300g  Level: T9-10 | Traumatic SCI  10 g impactor dropped from 25 mm. | - Sham + vehicle i.p. immediately, 8h, 24h post-surgery (n = 10) - Control, subject to SCI + vehicle i.p. immediately, 8h, 24h post-surgery (n = 10) - WIN 55,212-2 1 mg/kg i.p. immediately, 8h, 24h post-surgery (n = 10) - WIN 55,212-2 1 mg/kg i.p. immediately, 8h, 24h post-surgery + AM 251 1 mg/kg i.p. 30 min before each WIN 55,212-2 dose (n = 10) - WIN 55,212-2 1 mg/kg i.p. immediately, 8h, 24h post-surgery + AM 630 1 mg/kg i.p. 30 min before each WIN 55,212-2 dose (n = 10) | - BBB Locomotor score | - 1, 3, 7, 14, 21 and 28 days after surgery. | - Kruskal-Wallis test - Mann-Whitney U test | WIN,55212-2 treatment   - BBB scores in the control group reached a plateau 1 week after SCI, whereas BBB scores in the WIN,55212-2 treated continued to improve until 3 weeks after SCI when they scored more than 12. (Repeated measures ANOVA, Control vs WIN,55212-2, F = 38.49, p < 0.0001). - Comparison of WIN and Control animals at specific time points showed significant differences between the two groups at day 7, 14, 21 and 28; p < 0.01 at each individual timepoint.   AM 251 and AM 630 pre-treatment prior to WIN,55212-2 administration   - AM 630 pre-treatment significantly abrogated the BBB score improvement induced by WIN,55212-2, demonstrated by the discontinuation of functional recovery from 2 weeks after SCI (Repeated measures ANOVA, WIN vs WIN + AM 630 pre-treatment, F = 23.04, p = 0.0001). - Comparison of WIN and WIN + AM 630 pre-treatment groups at specific time points showed significant differences between the two groups at day 7, 14, 21 and 28; p< 0.01 at each timepoint. - No significant difference was noted between WIN and WIN + AM 251 pre-treated animals using repeated measures ANOVA; F = 1.873, p = 0.1879.   Comparison of WIN and WIN + AM 251 pre-treatment groups at specific time points showed significant difference between the two groups on day 28; p< 0.05 on day 28 only. |
| Su et al (2009) | n = 64  Male Sprague-Dawley  Age: Not stated  Weight: 300-350 g  Level: N/A | Ischemia/Reperfusion Injury  Aortic occlusion with Fogarthy catheter (tip at level of left subclavian artery) for 12 minutes. | - Control, right femoral artery isolated without occlusion, followed by SCI protocol (n = 8) - RIPC, 3 cycles of RIPC prior to SCI protocol (n = 8) - RIPC + AM251 1 mg/kg i.v. 15 min prior to RIPC before SCI protocol (n = 8) - RIPC + AM630 1 mg/kg i.v. 15 min prior to RIPC before SCI protocol (n = 8) - RIPC + vehicle i.v. 15 min prior to RIPC before SCI protocol (n = 8) - AM251 1 mg/kg i.v. 15 min prior to sham RIPC (n = 8) - AM630 1 mg/kg i.v. 15 min prior to sham RIPC (n = 8) - Vehicle i.v. 15 min prior to sham RIPC (n = 8)   Spinal cord I/R protocol was conducted 30 minutes after preconditioning or 45 minutes after chemical pre-treatment. | - 14-point motor deficit index (MDI) score | - 24 and 48 hours after reperfusion. | - Repeated measures analysis of variance (ANOVA) - Turkey’s multiple comparison test | - MDI scores of the RIPC treatment group were better than those of control (p < 0.05) and AM251 + RIPC groups (p < 0.01). - MDI scores of RIPC, AM630 + RIPC, and vehicle + RIPC treated groups were not significantly different. - MDI scores of control, AM251 + RIPC, AM251, AM630, and vehicle treated groups were not significantly different. |

# Supplementary File 6 – Risk of bias assessment

The Systematic Review Center for Laboratory Animal Experimentation (SYRCLE) Tool was used to evaluate risk of bias.

*Each item in the SYRCLE tool was scored as ‘Yes’, ‘No’, or ‘Unclear (?)’*

| **Bias assessment question** | Was the allocation sequence adequately generated and applied? (selection bias) | Were the groups similar at baseline or were they adjusted for confounders in the analysis? (selection bias) | Was the allocation adequately concealed? (selection bias) | Were the animals randomly housed during the experiment? (performance bias) | Were the caregivers and/or investigators blinded from knowledge which intervention each animal received during the experiment? (performance bias) | Were animals selected at random for outcome assessment? (detection bias) | Was the outcome assessor blinded? (detection bias) | Were incomplete outcome data adequately addressed? (attrition bias) | Are reports of the study free of selective outcome reporting? (reporting bias) | Was the study apparently free of other problems that could result in high risk of bias? (other bias) |
| --- | --- | --- | --- | --- | --- | --- | --- | --- | --- | --- |
| Ahmed et al (2010) | ? | ? | ? | ? | ? | ? | ? | Yes | Yes | Yes |
| Arevalo-Martin et al (2012) | ? | ? | ? | ? | ? | ? | ? | Yes | Yes | Yes |
| Genovese et al (2008) | Yes | ? | ? | ? | ? | ? | ? | Yes | Yes | Yes |
| Hama et al (2007) | ? | Yes | ? | ? | ? | ? | ? | Yes | Yes | Yes |
| Hama et al (2009) | ? | Yes | ? | ? | ? | ? | ? | Yes | Yes | Yes |
| Hama et al (2010) | ? | ? | ? | ? | ? | ? | ? | Yes | Yes | Yes |
| Hama et al (2011) | ? | ? | ? | ? | ? | ? | ? | Yes | Yes | Yes |
| Hama et al (2014) | Yes | ? | ? | ? | ? | ? | ? | Yes | Yes | Yes |
| Hong et al (2015) | Yes | Yes | ? | ? | ? | ? | Yes | No | Yes | Yes |
| Huo et al (2018) | Yes | ? | ? | ? | ? | ? | Yes | Yes | Yes | Yes |
| Impellizzeri et al (2017) | Yes | ? | ? | ? | ? | ? | Yes | Yes | Yes | Yes |
| Jing et al (2017) | Yes | ? | ? | ? | ? | ? | Yes | Yes | Yes | Yes |
| Kwiatkoski et al (2012) | Yes | Yes | ? | ? | ? | ? | Yes | Yes | Yes | Yes |
| Latini et al (2014) | ? | ? | ? | ? | ? | ? | ? | Yes | Yes | Yes |
| Li et al (2018) | Yes | ? | ? | ? | ? | ? | ? | Yes | Yes | Yes |
| Paterniti et al (2013a) | Yes | ? | ? | ? | ? | ? | ? | Yes | Yes | Yes |
| Paterniti et al (2013b) | Yes | ? | ? | ? | ? | ? | ? | Yes | Yes | Yes |
| Su et al (2009) | Yes | ? | ? | ? | ? | ? | Yes | Yes | Yes | Yes |
| Su et al (2017) | Yes | ? | ? | ? | ? | ? | Yes | Yes | Yes | Yes |

# Supplementary File 7 – Synthesis Without Meta-analysis (SWiM) reporting items checklist

| **SWiM reporting item** | **Item description** | **Page in manuscript where item is reported** | **Other*** |
| --- | --- | --- | --- |
| *Methods* | | | |
| **1** Grouping studies for synthesis | 1a) Provide a description of, and rationale for, the groups used in the synthesis (e.g., groupings of populations, interventions, outcomes, study design) | 8 |  |
|  | 1b) Detail and provide rationale for any changes made subsequent to the protocol in the groups used in the synthesis | N/A |  |
| **2** Describe the standardised metric and transformation methods used | Describe the standardised metric for each outcome. Explain why the metric(s) was chosen, and describe any methods used to transform the intervention effects, as reported in the study, to the standardised metric, citing any methodological guidance consulted | 8 |  |
| **3** Describe the synthesis methods | Describe and justify the methods used to synthesise the effects for each outcome when it was not possible to undertake a meta-analysis of effect estimates | 8 |  |
| **4** Criteria used to prioritise results for summary and synthesis | Where applicable, provide the criteria used, with supporting justification, to select the particular studies, or a particular study, for the main synthesis or to draw conclusions from the synthesis (e.g., based on study design, risk of bias assessments, directness in relation to the review question) | 8 |  |
| **5** Investigation of heterogeneity in reported effects | State the method(s) used to examine heterogeneity in reported effects when it was not possible to undertake a meta-analysis of effect estimates and its extensions to investigate heterogeneity | 8 |  |
| **6** Certainty of evidence | Describe the methods used to assess certainty of the synthesis findings | 8 |  |
| **7** Data presentation methods | Describe the graphical and tabular methods used to present the effects (e.g., tables, forest plots, harvest plots).  Specify key study characteristics (e.g., study design, risk of bias) used to order the studies, in the text and any tables or graphs, clearly referencing the studies included | 8 |  |
| *Results* | | | |
| **8** Reporting results | For each comparison and outcome, provide a description of the synthesised findings, and the certainty of the findings. Describe the result in language that is consistent with the question the synthesis addresses, and indicate which studies contribute to the synthesis | 10-21 |  |
| *Discussion* |  |  |  |
| **9** Limitations of the synthesis | Report the limitations of the synthesis methods used and/or the groupings used in the synthesis, and how these affect the conclusions that can be drawn in relation to the original review question | 27 |  |
